# Multi-set Pre-processing of Multicolor Flow Cytometry Data

**DOI:** 10.1038/s41598-020-66195-3

**Published:** 2020-06-16

**Authors:** Rita Folcarelli, Gerjen H. Tinnevelt, Bart Hilvering, Kristiaan Wouters, Selma van Staveren, Geert J. Postma, Nienke Vrisekoop, Lutgarde M. C. Buydens, Leo Koenderman, Jeroen J. Jansen

**Affiliations:** 1 Radboud University, Institute for Molecules and Materials, Analytical Chemistry, P.O. Box 9010, 6500 GL, Nijmegen, The Netherlands; 2 TI-COAST, Science Park 904, 1098 XH, Amsterdam, The Netherlands; 3 Department of Respiratory Medicine laboratory of translational immunology, University Medical Center Utrecht, Heidelberglaan 100, 3584CX, Utrecht, The Netherlands; 4 Department of Internal Medicine, Laboratory of Metabolism and Vascular Medicine, P.O. Box 616 (UNS50/14), 6200 MD, Maastricht, The Netherlands

**Keywords:** Translational immunology, Experimental models of disease, Cheminformatics

## Abstract

Flow Cytometry is an analytical technology to simultaneously measure multiple markers per single cell. Ten thousands to millions of single cells can be measured per sample and each sample may contain a different number of cells. All samples may be bundled together, leading to a ‘multi-set’ structure. Many multivariate methods have been developed for Flow Cytometry data but none of them considers this structure in their quantitative handling of the data. The standard pre-processing used by existing multivariate methods provides models mainly influenced by the samples with more cells, while such a model should provide a balanced view of the biomedical information within all measurements. We propose an alternative ‘multi-set’ preprocessing that corrects for the difference in number of cells measured, balancing the relative importance of each multi-cell sample in the data while using all data collected from these expensive analyses. Moreover, one case example shows how multi-set pre-processing may benefit removal of undesired measurement-to-measurement variability and another where class-based multi-set pre-processing enhances the studied response upon comparison to the control reference samples. Our results show that adjusting data analysis algorithms to consider this multi-set structure may greatly benefit immunological insight and classification performance of Flow Cytometry data.

## Introduction

Multicolor Flow Cytometry (MFC) is a powerful technique for quantitative detection of cellular marker expression at the single-cell level. MFC technology has become routine for biological studies and clinical diagnoses. In immunology, the main applications of MFC span the identification and quantification of cell subpopulations, monitoring of disease and its treatment and studying dynamic cellular processes such as cell differentiation^1–3^. Applications involve automated comparison of increasing numbers of samples, in which large numbers of cells are typically collected in every sample. Furthermore, many contemporary experiments quantitatively compare a ‘case’ (or responder) groups of samples against a ‘control’ (or healthy) sample group, in which expressions of identical cellular markers may be measured on the cells within all samples.

**Figure 1:**
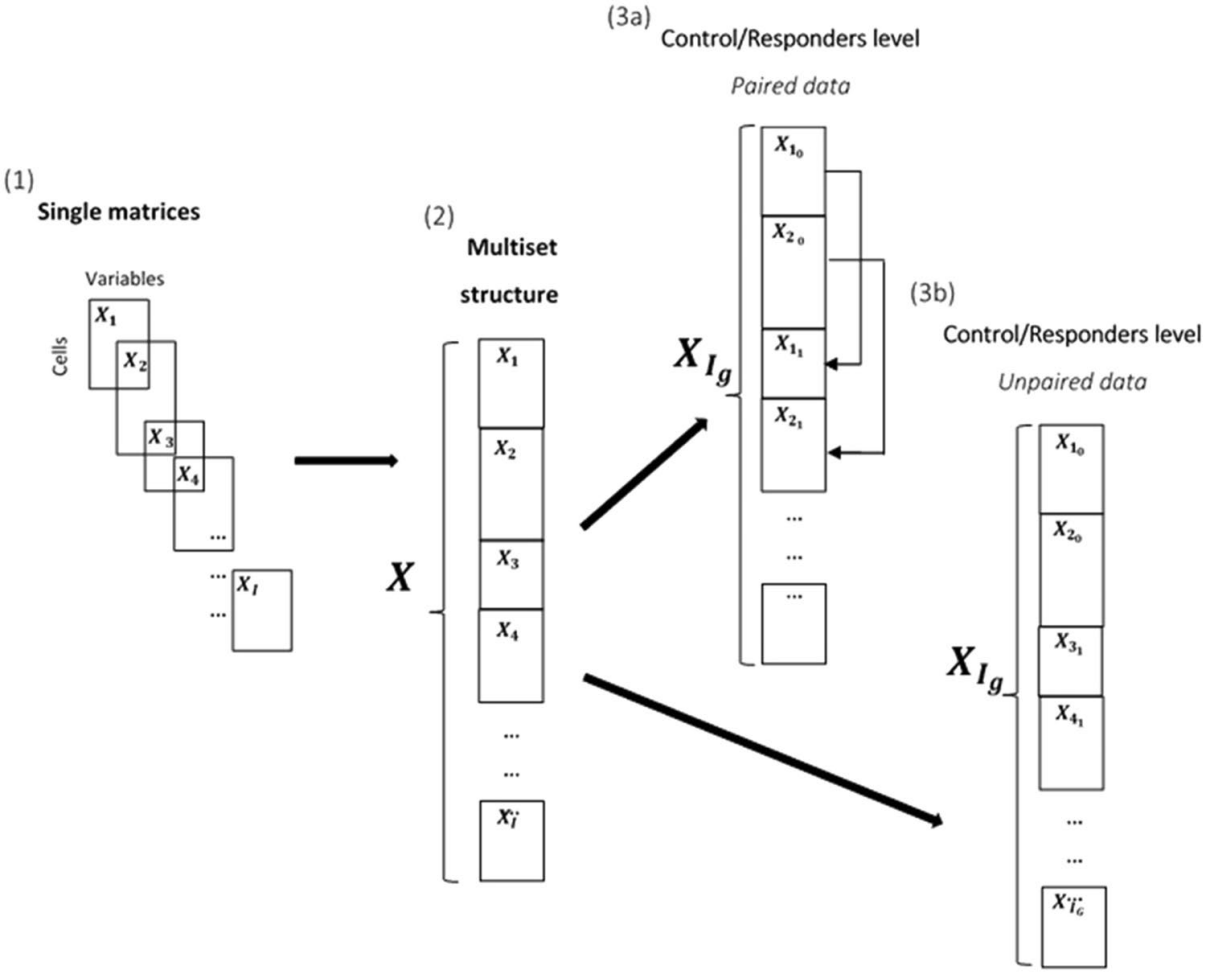
(**1**) Single data matrices representing measurement per sample, (**2**) When same variables are measured data can be arranged in a multi-set structure by linking the single matrices column-wise, (**3a**) Control/Responder differentiation of the multi-set structure, with paired data, (**3b**) Control/Responder differentiation of the multi-set structure, with unpaired data.

Figure 1 shows the possible arrangements of the MFC data by considering three different levels. Single matrices, which hold the cell set measured per sample (level 1); comprehensive analysis of different samples require that the same cellular markers are measured across all samples. Single matrices may then be concatenated column (or variable)-wise leading to a multi-set structure where each set contains the cells of one sample (level 2) as commonly described in chemometrics^4,5^. Each sample might be either a control or a responder and the information of the respective group is displayed in the level 3 of the multi-set structure. In some cases, samples are paired, which means that the same person is followed over time and analysed before and during an immune response. In this case the index i is not unique (3a); while for unpaired samples, each set is indicated by a different i (3b).

Several multivariate data analysis methods have been developed to quantitatively explore the cell composition of MFC samples^6^. To our knowledge, none of these methods quantitatively accounts for the multi-set structure of MFC data either in the pre-processing or in the analysis step. This may lead to a suboptimal overview and interpretability of the cell variability across all samples, instead reflecting non-biologically relevant inter-group or inter-sample variability. The problem especially occurs when different number of cells are measured between samples and/or baseline shifts are present between the samples due to e.g. technical variation.

Multivariate analysis methods such as Citrus^7^ and FlowSOM^8^ concatenate the MFC sample measurements in a single big matrix, without retaining the information about the different sets, prior to the analysis. Both methods then comprise mean-center and scaling based on such concatenated samples. If the various samples strongly differ in the number of cells measured, the calculated mean and standard deviation would be affected and, as result, the model will mainly describe the cellular marker variability of the samples with the most cells. Instead, a model where each sample equally contributes to, is desirable to avoid misleading results. In other algorithms, such as SPADE^9^ and viSNE^10^, problems related to difference in number of cells measured may be avoided by downsampling the data to a fixed and equal number of cells per sample. Downsampling causes loss of valuable data, which may result in an unreliable model estimation and may result in losing important cells that might be essential in several high-impact application of MFC, e.g. minimal residual disease detection.

The multi-set-structure has already been an integral part of the methods DAMACY^11^ and ECLIPSE^12^. Both DAMACY and ECLIPSE consider the multi-set structure during the pre-processing steps and when building the models to compare samples. However, until now we have not yet presented a detailed study to show how the multi-set pre-processing is in need when analysing MFC data, to avoid suboptimal immunological interpretation or even misinterpretation of the results.

In this paper we comprehensively review the multi-set structure and present the multiset pre-processing in order to create awareness for additional pre-processing options and how these options may be beneficial for the analysis and interpretation. The multi-set pre-processing corrects for the difference in number of cells measured per sample, by averaging the mean and the (square root) of the variance between samples and thus providing a more reliable representation of variability in the original marker expressions within the separate samples. We show how default pre-processing may be harmful by simulating a sample with fifty times more cells than other samples, by experimental quantification with an LPS benchmark study and with an obese versus lean study. The LPS study is based on neutrophils which has a high measurement-to-measurement variability and this unwanted technical variation affects all multivariate methods tested, namely PCA, SOM, t-SNE and Citrus. For this reason we show and advise to pre-process the data per sample, to correct for unwanted technical measurement-to-measurement variability and to highlight the studied underlying immunological effect. Pre-processing based on all cells of all control samples may enhance the deviation of immune response-specific marker variability from a control reference marker expression and lead to a better discrimination and diagnosis accuracy, as we show with DAMACY in an obese and lean study. Also algorithms such as viSNE and SPADE, which are distance based methods and do not explicitly use the mean, may benefit from the multi-set pre-processing. It may alter the relative differences between cells in different samples, thereby removing unwanted variation and essentially improving the information content of the models.

## Methods

Peripheral blood was extracted from subjects in both the LPS challenge study and obese versus lean study, all of whom gave their written informed consent before participating. All data were obtained using standardised protocols. The LPS challenge study and sample collection were approved by the medical ethics committee of Radboud University Medical Center (Radboudumc) Nijmegen, The Netherlands. The study protocol of the obese versus lean study was approved by the Medical Ethical Committee Jessa hospital, Hasselt, and Hasselt University, Belgium. Both studies were performed in accordance with the Declaration of Helsinki (Forteleza, 2013).

### LPS study data

MFC data of the “Lipopolysaccharide study” were part of an endotoxin trial (NCT01374711; www.clinicaltrials.gov), in which male healthy donors were challenged with intravenous administration of Lipopolysaccharide (LPS). The LPS dataset comprises gated neutrophils from 16 samples: 8 ‘control’ (or reference) samples who did not receive LPS, and 8 different ‘response’ samples who were administered with LPS. For the responders, whole blood was collected 180 minutes post LPS administration. Seven surface markers were measured on the neutrophils in samples of both classes: CD62L, CD11b, CD11c, CD64, CD32, CD69, and CD16. The samples size range between 6 thousand to 40 thousands cells. Further details regarding the Flow Cytometry experiments that generated the data can be found in a previous publication^13^.

### Obese data

The obese data comprises 29 samples: 13 lean samples with Body mass index (BMI) in the range between 20.83 and 25.62 and 16 obese samples with BMI between 30.47 and 49.27^14^. The following markers were measured: CXRCR1, CD14, CD56, CD11b, CD11c, CD16, HLA-DR, CD3/CD19/CD66b. The data was gated on innate cells by removing all cell positive for markers CD3, CD19, CD66b. The samples have 2 thousand to 24 thousand cells left after gating.

### Data pre-processing

Data pre-processing is a crucial aspect of multivariate data analysis^15^ to remove variability in the data that is unrelated to the problem under study, while retaining the experimentally relevant information. In Flow Cytometry, such irrelevant variability might result from instrumental artefacts due to misalignment of the laser source, baseline drift, laser power variability, or uninformative noise coming from low intensity signals. Time delays between sample collection and measurements can also bring variability that is not related to the problem under study. Especially granulocytes should either be measured fresh and as fast as possible, or measured after using advanced freezing techniques to minimize such nonspecific activation^16,17^.

MFC raw data can be arranged in the matrix $$\text{X}\,=\,[\begin{array}{c}{\text{X}}_{1}\\ \vdots \\ {\text{X}}_{I}\end{array}]$$ of size $\mathop{\sum }\limits_{1}^{I}{N}_{{i}_{g}}\text{x}J$, where ${N}_{{i}_{g}}$ is the number of cells of the *i*_*g*_^th^ sample, with *g* = 0 used for the control group and *g* ≥ 1 for responder groups and *J* corresponds to the markers measured, 1…*j*…*J*. The first step of the pre-processing consists of transforming **X** with log (Eq. 1a) or hyperbolic inverse sine (arcsinh) function (Eq. 1b)^18^.

1a $${{\bf{X}}}_{\text{log}}={\text{log}}_{10}({\bf{X}})$$

1b $${{\bf{X}}}_{\text{log}}\,=\,\text{log}\,(\,\frac{\text{X}}{\text{c}}+\sqrt{{\left(\frac{\text{X}}{\text{c}},+,1\right)}^{2}})$$

These transformations perform a non-linear conversion of the data and they are generally applied to correct for heteroscedasticity and to change skewed distributions into more symmetric, Gaussian distributed peaks. A log transformation may be still used on datasets exported with only positive data values, arcsinh transformation has been introduced to accommodate for the negative values in MFC data that may result from the background subtractions performed by newer digital MFC technology, or by compensation^19^. We used arcsinh transformation with a default cofactor c value of 150 and used visual inspection of the data to preclude the emergence of any ‘split peaks’ upon transformation^20^.

### Multi-set centering and scaling

Arcsinh and log transformation can be considered as a ‘pseudo scaling’ transformation that ameliorates magnitude differences in the fluorescence emissions *per* fluorophore between different markers. However, full removal of such differences requires variable scaling and mean centering after transformation^21^. Mean (or median) centering subtracts the column mean (or median) from every element in the column. Median centering is required when the number of cells measured is very low. This removes marker expression (or offsets) consistently present across all the cells and creates a common point of reference to quantify variability in cellular marker expression between the cells. Centering is typically applied in combination with scaling, which consists in dividing each variable by a scaling factor. Scaling equalizes the variability of each cellular marker across the cells. This allows the variability in every surface marker to contribute equally to a multivariate model of the data, regardless of the intensity of the used fluorophore or the absolute variability in abundance of every surface marker.

In the pre-processing as applied in Citrus^7^ and flowSOM^8^ analyses, all the data files are bundle together in a big data matrix and, after transformation, centering is performed according to Equation 2:

2a $${\bf{m}}=\frac{1}{N}{1}_{N}^{{\bf{T}}}{{\bf{X}}}_{\text{log}}$$

2b $${{\bf{X}}}_{{\bf{m}}}={{\bf{X}}}_{\text{log}}-{1}_{N}{\text{m}}^{\text{T}}$$

where **m** is the mean of the arcsinh (or log-) transformed cellular marker expression calculated across all the samples included in the matrix **X**_log_; *N* is the number of total cells measured; **1** is a column vector of ones with length *N*. The mean-centered matrix **X**_**m**_ is then scaled according to Equation 3:

3a $${{\bf{s}}}^{\text{T}}=\sqrt{var({{\bf{X}}}_{{\bf{m}}})}$$

3b $${\bf{S}}={\bf{d}}{\bf{i}}{\bf{a}}{\bf{g}}({{\bf{s}}}^{\text{T}})$$

3c $${{\bf{X}}}_{\text{sc}}={{\bf{X}}}_{{\bf{m}}}{{\bf{S}}}^{\text{T}}$$

with **S** diagonal matrix holding the standard deviation **s**^T^; **X**_sc_ the resulting auto-scaled matrix.

This ‘standard’ pre-processing ignores the multi-set structure (Fig. 1) of multi-cells Flow Cytometry data which means that the information of cells belonging to a specific sample or group is lost.

Our ‘multi-set’ pre-processing specifically accommodates the multi-set structure of Flow Cytometry data and tackles various sub-aspects: cells measured may belong to different samples, where these can be drawn from different experimental cohorts (such as control and responder) and different numbers of cells can be measured per sample. Several strategies for centering and scaling^21^ are therefore available which may lead to different views and information: to centre/scale using the mean/standard deviation calculated on all the samples, on the control samples or per sample.

The different pre-processing strategies were tested on simulated data which consisted of normal distributions, representing different cell populations (Online Supplementary Material I).

**Figure 2:**
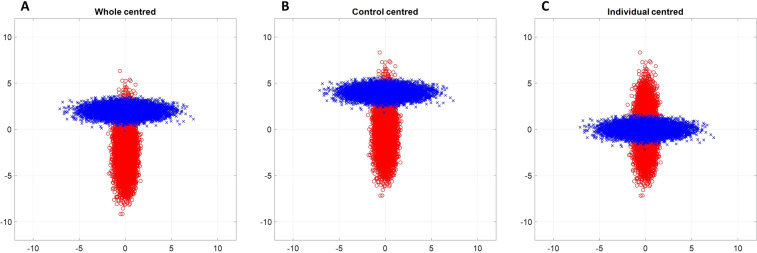
2D scatter plot of the simulated data after applying different types of centering on both control (red) and responder (blue) populations. Left (**A**) data are centered using the mean calculated on the whole dataset and correcting for differences in # cells measured per sample; Center (**B**) data are centered using the mean estimated for the control samples and correcting for differences in # cells measured per control; Right (**C**) data are mean center per sample.

In Fig. 2 the effect of three different types of centering on this dataset is displayed, without any scaling: centering over the whole dataset, based on group-level, and per sample.

Centering over the whole dataset employs the data from both the responder and control groups. This centering operation requires a correction for the possibly different number of cells measured per sample, to avoid the sample with most cells dominating the calculated mean. Thus, creating a common reference point between the samples and not in the sample with the most cells, see for more infomration supplementary Figure S3, S5B and S7B. This is done to equally weighting each sample in the calculation of the overall mean, according to Eq. 4:

4a $${{\bf{m}}}_{{i}_{g}}=\frac{1}{{N}_{{i}_{g}}}{1}_{{N}_{{i}_{{i}_{g}}}}^{{\bf{T}}}{{\bf{X}}}_{\text{log},{i}_{g}}$$

4b $${\bf{m}}=\frac{{\sum }_{{\boldsymbol{g}}=1}^{{\boldsymbol{G}}}{\sum }_{{{\boldsymbol{i}}}_{{\boldsymbol{g}}}=1}^{{{\boldsymbol{I}}}_{{\boldsymbol{g}}}}{{\bf{m}}}_{{i}_{g}}}{{\sum }_{g=1}^{G}{I}_{g}}$$

4c $${{\bf{X}}}_{{\bf{m}}}={{\bf{X}}}_{\text{log}}-{1}_{{N}_{{i}_{g}}}{{\bf{m}}}^{\text{T}}$$

Here, the average marker expression **X**_**m**_ based on all the samples **m** is calculated by dividing the sample-specific mean ${{\bf{m}}}_{{i}_{g}}$, estimated per each sample *i* of group *g*, over the total number of samples *I*_*g*_. Centering over the whole dataset (Eq. 4c) translates the means of both groups around the axis coordinate origin (*m*_*control*_ = 0,−2; *m*_*response*_ = 0,2), as displayed in Fig. 2a.

Centering can be performed based on group (control)-level, which means the control group is used as point of reference. Centering based on controls is given by equation:

5a $${{\bf{m}}}_{0}=\frac{{\sum }_{{1}_{0}}^{{I}_{0}}{{\bf{m}}}_{{i}_{0}}}{{I}_{0}}$$

5b $${{\bf{X}}}_{{\text{m}}_{0}}={{\bf{X}}}_{\text{log}}-{1}_{{N}_{{i}_{g}}}{\text{m}}_{0}^{\text{T}}$$

Also in this case, a correction for different numbers of cells per sample is performed by using the mean of each *i*_0_–th control sample ${{\bf{m}}}_{{i}_{0}}\,$to calculate the weighted control class mean **m**_0_, where *I*_0_ represents the number of control samples. The resulting ${{\bf{X}}}_{{\text{m}}_{0}}$, of size ${N}_{{i}_{0}}\text{x}J$, represents the multi-set matrix centered using the class mean of the log-transformed surface marker intensities of the control group. Centring based on the control samples (Eq. 5b) will remove the shift of the control cells of which variability is used as reference. This emphasizes the deviation of responder cell variability (*m*_*response*_ = 0,2) from the control reference (*m*_*control*_ = 0,0), as shown in Fig. 2b.

Centering per multi-cell set, *i.e. per* MFC sample, is calculated as follows:

6a $${{\bf{m}}}_{{i}_{g}}=\frac{1}{{N}_{{i}_{g}}}{1}_{{N}_{{i}_{{i}_{g}}}}^{{\bf{T}}}{{\bf{X}}}_{\text{log},{i}_{g}}$$

6b $${{\bf{X}}}_{{{\bf{m}}}_{{i}_{g}}}={{\bf{X}}}_{\text{log},{i}_{g}}-{1}_{{N}_{{i}_{g}}}{\text{m}}_{{i}_{g}}^{\text{T}}$$

where ${{\bf{m}}}_{{i}_{g}}$ is the mean calculated for the cellular markers measurements of the *i*_*g*_–th sample, ${i}_{g}={1}_{g},\ldots ,{\text{I}}_{\text{G}}$, the matrix ${{\bf{X}}}_{\text{log},{i}_{g}}$ of size ${N}_{{i}_{g}}\text{x}J$; ${N}_{{i}_{g}}$ corresponds to the number of cells of the *i*_*g*_–th sample; **1** is a column vector of ones with length $\,{N}_{{i}_{g}}$. Centering *per* sample (or *per* individual) (Eq. 6b) removes the shift per sample in both groups (*m*_*control*_ = *m*_*response*_ = 0,0), as shown in Fig. 2c. This strategy may be used to correct for technical sample-specific offsets due to *e.g*. changes and/or misalignment of laser intensity, sample handling etc. that are unrelated to the biomedical information within a MFC dataset.

**Figure 3:**
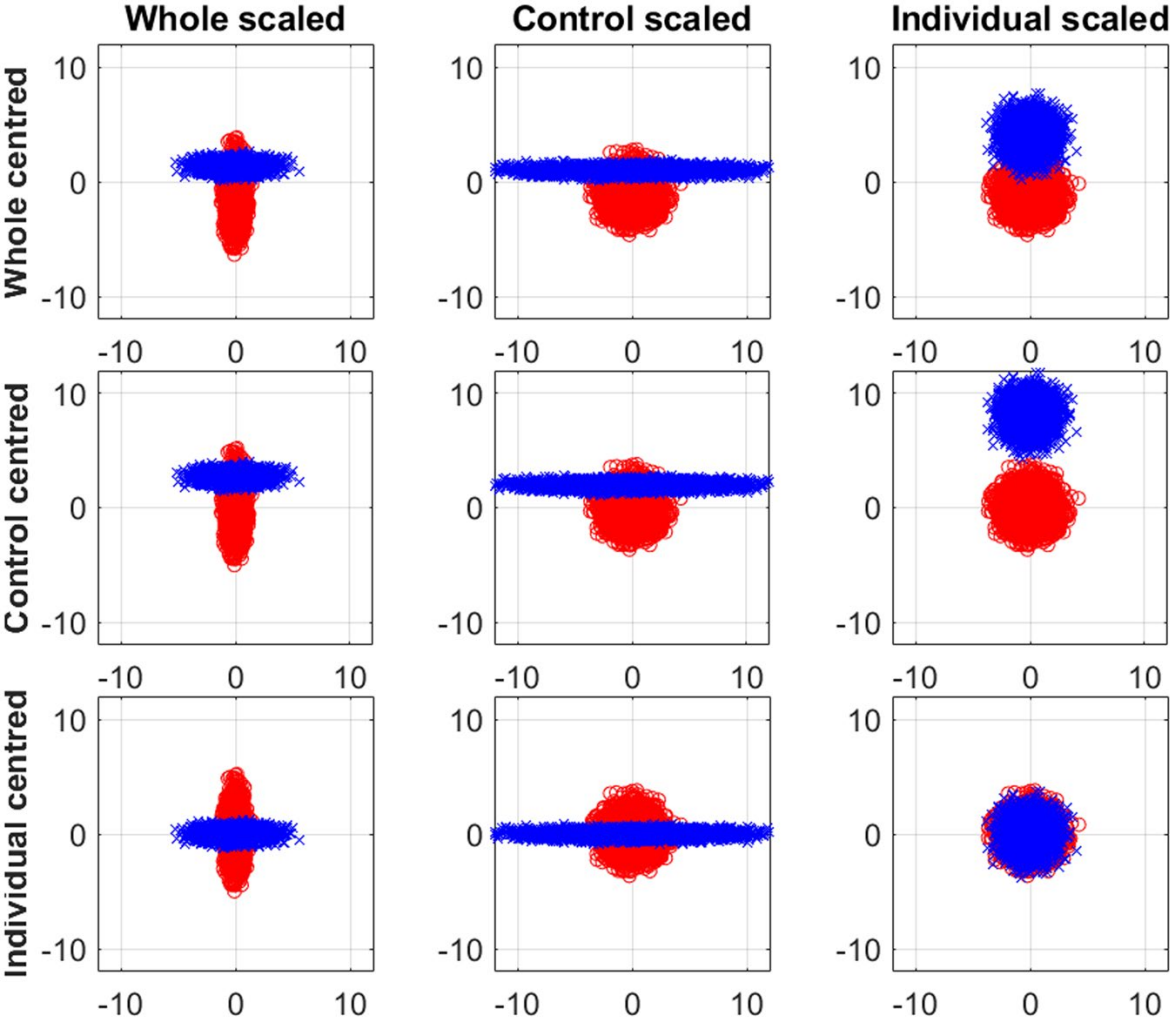
2D scatter plot of the simulated data pre-processed with different pre-processing options. A ‘control’ population (red rounds) and a ‘responder’ population (blue triangles) are present. The columns display the scaling options (from left to right): scaling over the whole dataset, scaling based on the control group and scaling per sample. The rows correspond to the centring options (from top to bottom): centering over the whole dataset, centering based on the control group and centring per sample.

Additionally to centering, scaling is performed. As for centering, the same alternatives are available also for the scaling step. Below, we discuss and show the formulas of the different scaling options performed on the sample mean centered data matrix ${{\bf{X}}}_{{{\bf{m}}}_{{i}_{g}}}$ (Eq. 4). Each formula can be easily adapted to the other types of centering. Similar conclusions about the effect of scaling can be drawn and they are summarized in Fig. 3.

Scaling over the whole dataset is performed with the flowing equation:

7a $${{\bf{s}}}^{{\bf{T}}}=\sqrt{\frac{{\sum }_{g=1}^{G}{\sum }_{i=1}^{{I}_{g}}var({{\bf{X}}}_{{{\bf{m}}}_{{i}_{g}}})}{{\sum }_{g=1}^{G}{I}_{g}}}$$

7b $${\bf{S}}={\bf{d}}{\bf{i}}{\bf{a}}{\bf{g}}({{\bf{s}}}^{{\bf{T}}})$$

7c $${{\bf{X}}}_{{\bf{s}}{\bf{c}}}={{\bf{X}}}_{{{\bf{m}}}_{i}}{{\bf{S}}}^{-1}$$

where **S** is a diagonal matrix of size *J* × *J* containing the standard deviation (or median absolute deviation) of each surface marker **s**^**T**^, calculated on all cells of all samples. Median absolute deviation is required when the number of cells measured is very low. Scaling based on the complete data will keep the same difference in shape between control (*s*_*control*_ = 0.34, 1.37) and responder (*s*_*response*_ = 1.37, 0.34) population, but the variables are now equally important (*s* = 1,1).

Scaling based on the control group is given in Eq. 8:

8a $${{\bf{s}}}_{0}^{{\bf{T}}}=\sqrt{\frac{{\sum }_{i=0}^{{I}_{0}}var({{\bf{X}}}_{{{\bf{m}}}_{i}})}{{I}_{0}}}$$

8b $${{\bf{S}}}_{0}={\bf{d}}{\bf{i}}{\bf{a}}{\bf{g}}({{\bf{s}}}_{0}^{{\bf{T}}})$$

8c $${{\bf{X}}}_{{\bf{s}}{\bf{c}}}={{\bf{X}}}_{{{\bf{m}}}_{i}}{{{\bf{S}}}_{0}}^{-1}$$

With **S**_0_ of dimensions (*J* × *J*) with the diagonal element containing the standard deviation ${{\bf{s}}}_{0}^{{\bf{T}}}$ of each surface marker based on all cells of control samples. Scaling based on the control group will remove the shape of the control (*s*_*control*_ = 1, 1) and emphasize the shape of the challenged population (*s*_*response*_ = 4, 0.25).

Scaling per sample is calculated as follows:

9a $${{\bf{s}}}_{{i}_{g}}^{{\bf{T}}}\,=\sqrt{var({{\bf{X}}}_{{{\bf{m}}}_{i}})}$$

9b $${{\bf{S}}}_{{i}_{g}}={\bf{d}}{\bf{i}}{\bf{a}}{\bf{g}}({{\bf{s}}}_{{i}_{g}}^{{\bf{T}}})$$

9c $${{\bf{X}}}_{\text{sc}}={{\bf{X}}}_{{{\bf{m}}}_{i}}{{\bf{S}}}_{{i}_{g}}^{-1}$$

where ${{\bf{S}}}_{{i}_{g}}$ is diagonal matrix of size *J* × *J* holding the standard deviation of the mean-centered surface markers of the *i*_*g*_–th sample. Scaling per sample makes cell population of both groups homogenous (*s*_*control*_ = *s*_*response*_= 1, 1).

The plots in Fig. 3 show that, the method of pre-processing may greatly determine the pre-processed data structure. Centering and scaling based on the control class will enhance the deviations of the responder samples from the cell variability observed in the control samples. Alternatively, sample centering and scaling might be a preferable option when measurements were influenced by differences in the (technical or practical) experimental procedure per sample. However, it should be noted that the last option has a considerable disadvantage. When all cells of one response sample show up or downregulation of one or multiple markers compared to the cells of the control samples, this information will be lost due to sample centering and scaling. However, when the technical differences are bigger than the biological differences and thus the between sample variability is higher than the between group variability, it may be the only solution as the shape and number of cells may still be important, see supplementary Scheme S1.

### Correcting the number of cells per sample for principal component analysis

Principal Component Analysis (PCA) is a widely used method to visualize multidimensional data, including Flow Cytometry data, while retaining most of the variability expressed in the originally measured variables^22,23^. A multi-set extension of PCA exists to accommodate multi-set structure present in the data and it is known as Simultaneous Component Analysis (SCA)^24^. When applying the SCA decomposition, the pre-processed matrix **X**_sc_ is normalized such that each sample contributes with the same amount of information. The normalization is done by blockscaling^25^, which consists of dividing each sample by the square root of the corresponding number of cells, see Eq. 10:

10 $${{\bf{X}}}_{\text{sc}}^{\ast }=[\begin{array}{c}{{\bf{X}}}_{{\bf{s}}{\bf{c}}{1}_{1}}{N}_{11}^{-1/2}\\ \vdots \\ {{\bf{X}}}_{\text{sc}{I}_{G}}{N}_{IG}^{-1/2}\end{array}]$$

SCA then decomposed the resulting matrix as follows:

11a $${{\bf{X}}}_{\text{sc}}^{\ast }={{\bf{T}}}_{\ast }{{\bf{P}}}_{\ast }^{{\bf{T}}}+{\bf{E}}$$

11b $${\bf{T}}={{\bf{X}}}_{\text{sc}}{{\bf{P}}}_{\ast }$$

where **T** of size $\mathop{\sum }\limits_{{i}_{g}={1}_{1}}^{{I}_{G}}{N}_{{i}_{G}}\times K$ contains the SCA scores, *k* = 1,…, *K* indicates the dimensionality of the new low-dimensional space, P_*_ of size *K* × *J* containing the loadings and **E** being the residuals. The loadings represent the contribution of each cellular marker in building the low dimensional space and in describing the cell variability contained in the scores matrix. Blockscaling is essential to estimate loadings which are not highly influenced by the cellular maker variability of the samples with the most samples. The relations between cell variability and the related expression of markers in the SCA space may be combined in a single biplot^26^.

## Results

### The effect of measurement-to-measurement variability and dedicated pre-processing on the interpretation of viSNE, SOM and Citrus models of real life data: the Lipopolysaccharide study

The Lipopolysaccharide (LPS) challenge study entails intravenous administration of systemic endotoxin to eight volunteers. This experiment is used to mimic acute inflammation in humans which is most prominent 180 minutes after LPS administration^13,27^. Previous publications have shown that upon acute inflammation, two neutrophil subsets arise in the peripheral blood, which differentially express FcγRIII (CD16) and L-selectin (CD62L). Neutrophils under homeostasis express both CD16 and CD62L, while both arising subsets are CD16+CD62L- and CD16-CD62L+, with different morphological and functional features^13^. The challenge aimed to characterize the expression of activation markers on the neutrophil subsets released in peripheral blood during the LPS-induced response, compared to a homeostatic reference present in the control group. Considerable shifts of the fluorescence signals are present between the samples in both groups for nearly all markers, as shown in Figure S8 in the Supplementary Material II. Changes in the absolute position of the same cell population across the samples might result from both (not-relevant) biological and technical variation^28^. Various studies have shown how different sample treatments may affect marker expression on neutrophils^16,17^. Additionally, time delay between sample collections, reagent staining and actual measurements may vary across the samples and this introduces an uncontrolled between-sample variation which can hinder an accurate data analysis and influence interpretation of the findings when the data is not accurately corrected for this.

After log-transformation, we pre-processed the LPS data with both standard pre-processing, according to Equations 1–2, and multiset pre-processing, consisting of mean-centering per individual and scaling over the control group (Equations 6–8). This multi-set pre-processing accounts for difference in terms of numbers of cells measured per individual sample and may correct for shifts caused by between-sample variability.

**Figure 4:**
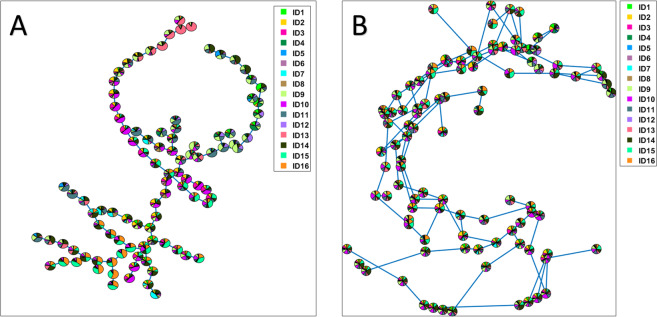
SOM analysis results. Nodes of the SOM trees are colored according to the number of cells belonging to the different individuals. Panel (**A**) SOM tree results obtained for the standard pre-processed LPS dataset, consisting of centering and scaling by using mean and standard deviation calculated over the all the samples; Panel (**B)** SOM tree results obtained for the multi-set pre-processed LPS dataset, consisting of centering per individual and scaling over the control individuals.

The effect of the different pre-processing strategies on the LPS data was investigated by applying self-organizing (SOM) map using the toolbox implemented in Matlab^29^ with same parameter settings as in flowSOM^8^: grid size of 10 ×10, Euclidean distance to find nearest neighbor, and training length of 10 epochs. The resulting clustering performed by SOM trained on the standard pre-processed and multi-set preprocessed data can be visualized in the Supplementary Figure 9A-B, respectively. Nodes are displayed with pie chart representing the average intensities of the markers for all the cells assigned to the specific node. Based on this representation, we can observed that both trees seem to be dominated by the majority of normal mature neutrophils having CD16+CD62L+ expression. Previous work has shown how the vast number of normal-like cells presents in the responder individuals can hamper the identification of response-specific cell subsets^12^. However, when coloring the pie chart according to the number of cells from each sample in the nodes, a major difference is revealed between the clustering results for the diversely pre-processed data (Fig. 4A-B, respectively). A sample-specific clustering is present in the SOM tree obtained for the standard pre-processed data (Fig. 4A). In fact, a considerable number of nodes consists of cells from mainly the same sample or only a few samples. This indicates that SOM algorithm is influenced by between-sample variability which dominates the model at the expenses of the subtler variability related to the homogenous LPS-induced response.

Such between-samples variability is removed by the multi-set pre-processing and as a result the cells from the 16 samples are distributed throughout the tree (Fig. 4B). The multi-set pre-processing thus leads to a cell clustering mostly based on marker expression variability within the samples, rather than between samples.

An insight at single-cell resolution level was obtained by viSNE^10^ analysis. The analysis was done using the Matlab GUI *cyt*, downloaded from the website https://www.c2b2.columbia.edu/danapeerlab. For each sample we randomly selected a subset of 2000 cells, so that the total number of cells analyzed was 32.000. The results of viSNE analysis are shown in the Supplementary Material II (Figure S10). Likewise for the SOM representation, when performing viSNE on the standard pre-processed data, cells of the same sample are mostly grouped together (Figure S10A), suggesting that the clusters found by the algorithm are sample specific. When the viSNE map is colored according to the control/response group (Figure S10B), cells of the control and response samples overlap considerably. The upper left region and the middle low area appear to be distinctive regions for the responder. However, not all the responder samples show cells in those regions. Single marker expression profiles are visualized in Figure S11. Premature (banded nucleus) neutrophils can be detected as having a typical CD16-CD62L+ expression. We therefore gated the region corresponding to this phenotype in the viSNE map and extracted the cells within the gate. Cells contained in the gate, and thus associated to the premature phenotype, are mainly represented by cells from a single sample (Patient #5), as shown in the bar plot Figure S12 of the Online Supplementary Material II.

Contrary to the standard pre-processed data, the viSNE map obtained for the multi-set preprocessed data, colored per sample, shows how the cells are distributed across the map and no sample-specific clusters are present (Figure S14A). As expected, considerable overlap is present between cells from the responder (red, Figure S14B) and control groups (blue, Figure S14B).The map is then colored according to the seven marker expression profiles. LPS-specific cells CD62L-CD16+CD11b+ are well distinct in the upper left part of the map (Figure S15). Pre-mature neutrophils CD16-CD62L+ were also identified. As done for the previous viSNE analysis we gated this region and we observed that cells within the gate are from multiple responders as shown in the bar plot Figure S16. The viSNE analysis applied on the multi-set pre-processed data thus models the LPS-induced response across all the responder individuals and no sample-to-sample variation seems to be dominant. Thus multiset pre-processing better reflects the original data where all responders showed a broader CD16 distribution compared to controls, see Figure S8.

In order to experimental quantify whether the pre-processing could affect the results of a discrimination model between the control and responder groups, we performed a Citrus^7^ analysis on the Cytobank platform (https://www.cytobank.org/). The model identified as optimal for the analysis on the standard pre-processed data (cv.min in Fig S16A, Supplementary Material II) provided the highest accuracy achievable, corresponding to 25% of misclassified samples. When Citrus analysis was trained on the multi-set pre-processed data, a perfect classification was obtained as shown in the Supplementary Material II (Fig S17A). The phenotypes of the cell clusters associated with the four features, corresponding to a null cross-validation error rate, are shown in Fig S17B. The first three clusters are more abundant in the responder group and they may be associated to premature and mature neutrophils. The last cluster found more present in the control group compared to the responder group may be assigned to normal mature neutrophils having CD16+CD62L+ expression.

### Experimental quantification of correcting for sample size and multiset pre-processing using Discriminant Analysis of Multi-Aspect Cytometry (DAMACY)

In this section we used Discriminant Analysis of Multi-Aspect Cytometry (DAMACY)^11^ to explore the different pre-processing options mentioned in the method section. DAMACY^11^ first describes the cellular variability in N-dimensional histograms based on Simultaneous Component Analysis (SCA) of the pre-processed data using the multi-set structure. Subsequently it uses Orthogonal Partial Least Squares Discriminant Analysis (OPLS-DA)^30^ on the histograms to create a regression map. The regression map shows which cell (sub)populations are more or less present in a clinical phenotype compared to control samples. The whole algorithm, including the pre-processing step, was validated using leave one out validation in the LPS results and seven-fold cross-validation with fifty iteration in the obese dataset. Both datasets were permutated 1000 times for permutation testing. This leads to well statically validated prediction accuracies that may be compared with each other and compared to maximum accuracy achieved on the permutated data to see if the models are significant and not by chance have a high accuracy. Only DAMACY was used, because the multi-set preprocessing options are not yet incorporated in other methods and thus unable to correctly validate the results. The only multiset pre-processing option possible in other methods such as Citrus (previous paragraph) is individual centering and scaling since those are not affected by the cross validation.

### LPS results

In addition to providing enhanced insight, multi-set pre-processing may also benefit the discriminative power between control and LPS responders. Supplementary table 1 shows the prediction performance of different pre-processing strategies using DAMACY. Standard concatenating the data and subsequently autoscaling leads to a prediction accuracy of 75% and a p-value of <720/1000. Perfect prediction (p-value <4/1000) is acquired when multi-set individual centering combined with scaling over the entire set and correcting for the number of cells per sample or scaling per individual sample. Also the number of orthogonal latent variables (OLV) drops, probably because the pre-processing already removed a large part of the orthogonal information. Centering based on the whole set in combination with scaling per individual sample leads to an unpredictable model. The data is characterized by shifts in mean due to technical variation of the samples, but also has a biological increase in variability in most markers due to the LPS induced effect. Scaling per individual without centering per individual only removes biological but not the technical variability, which therefore lowers prediction accuracy.

### Obesity versus lean data

Different pre-processing strategies were tested with DAMACY on the obese vs lean dataset, using seven-fold cross validation with 50 repetitions. The prediction performance is summarized in supplementary table 2. Standard pre-processing of the data leads already to a high prediction accuracy of 76.6% (p-value < 28/1000), mainly because the obese samples are well predicted (high sensitivity). The obese samples have more cells measured compared to the lean individuals and are therefore more important in the model and also better predicted. Centering and scaling based on the whole set considering the number of cells per sample (dark blue) creates a model with worse accuracy but with increased specificity. Centering based on the controls (lean individuals) enhanced the difference between obese and lean and led to a slightly better model than standard pre-processing. Individual centering of the data removes the shift in marker expression found in obese individuals and results in bad predicting models, while individual scaling in this data improved the prediction accuracy.

**Figure 5:**
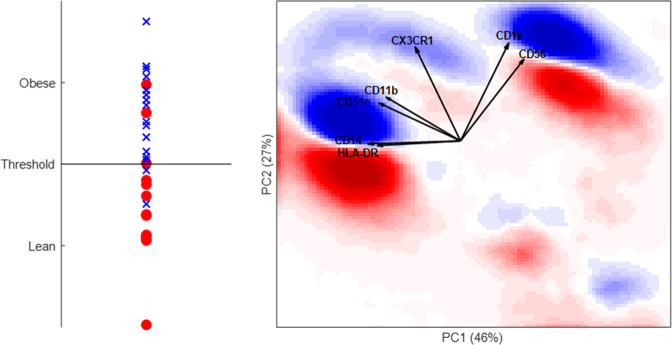
DAMACY model of obese versus lean data with optimal centering based on control and scaling per individual. The left panel shows the average prediction score of the OPLS-DA model of controls as red rounds and asthma individuals as blue crosses. The right panel shows negative weights as red and positive weights as blue. The loadings of the Base model are plotted on top as black vectors and indicate how each surface marker contributes to the cell variability in a specific direction within the model.

Figure 5 shows the DAMACY model based on optimal pre-processing, while DAMACY model based on standard pre-processing and worst pre-processing are displayed in Figure S19 and Figure S20 of the Online Supplementary Material III, respectively. Cells identified as classical monocytes are in direction of CD14 and HLA-DR and they show a split in upper blue highlighted and lower red highlighted area which corresponds to increasing CD11b, CD11c and CX3CR1 expression in cells more present in obese individuals. The same increase is also observed in NK cells in direction of CD16 and CD56. Moreover, a trend in blue highlighted areas corresponding to more cells in obese individuals from CD14 towards CD16 is observed, which corresponds to transition of classical, intermediate and non-classical monocytes (CD14−CD16+). The same information is more difficult to extract from the model based on standard pre-processing, because the trend is more skewed, see Figure S19 and completely absent in the model based on worst pre-processing, see Figure S20. Therefore, multi-set pre-processing is beneficial for interpretation, even when it does not specifically enhance diagnostic potential. However a screening through all the possible options should be done in order to obtain the optimal results^15^.

## Discussion

The integration of the multi-set structure in the pre-processing and multivariate analysis steps of MFC data is essential to overcome the influences of non-informative aspects in the data. Here we showed that our multi-set pre-processing may be essential to extract the full biomedical information from MFC data consisting of samples (sets) which may belong to different groups (*e.g*. case *vs* control) and may be characterized by different number of cells measured. Based on classification accuracy, biomedical insight or both.

### Difference number of cells measured per set

If the difference in number of cells measured between samples is not corrected, this may bias the analysis results and thus lead to a misleading interpretation of the findings. This happens because when MFC data are pre-processed (by mean centering and scaling), as standardly done in most analysis methods such as Citrus and flowSOM, the calculated mean shifts towards the sample(s) with most cells (see simulated data results). The effect of the shift in multivariate analysis is shown by Simultaneous Component Analysis (SCA), which was chosen as it offers a representation of cells population together with marker (co)-expression in a single plot. Blockscaling (Eq. 11) is an integral part of the SCA analysis when estimating the loadings. The SCA scores are then calculated by multiplying the pre-processed data with such corrected loadings. We showed that only when blockscaling is combined with multi-set pre-processing, the resulting SCA model offers the best representation of the original marker expression when the number of cells in one of the samples was increased (Figure S7). In other simulated data (Figure S5) with less correlation structure because only three variables were measured, blockscaling had a negligible influence but the correction provided by the multi-set pre-processing improved the interpretation of the results. The correction provided by our multi-set pre-processing can be a more optimal solution to down-sample the sets that uses all collected data, as done for SPADE and (optionally) in viSNE, to make each sample equally contribute to the built model. Additionally, it will be crucial when low abundant cell populations are relevant, as their detection could be hampered as the mean shifts greatly to the more abundant populations.

### Measurement-to-measurement variability

Finding biological variability in the samples which is relevant to the studied response/disease is one of major challenge in MFC data analysis. Our multi-set pre-processing strategy enables to systematically remove variability unrelated to the studied problem while retaining the informative biological information. Non-relevant biological or technical variation could provoke shifts of fluorescence signals of the same cell population among the samples, as in the case of the LPS dataset. When applying standard pre-processing to the LPS data, this variability is not removed and remained quite dominant in the multivariate analyses performed. The viSNE and SOM models thus intrinsically described mainly this unwanted variability leading to trivial conclusions. In fact, the obtained results enabled us to identify putative phenotypically different cells subsets which are not realistically representing the homogenous immune response across all the samples. We showed how this sample-to-sample variability might also lead to a suboptimal discrimination accuracy in Citrus and DAMACY analysis. By using multi-set pre-processing was beneficial for viSNE and SOM analyses of which results were better representing the phenotypical variation present in the data. It also helped in outperforming the predictive ability of the Citrus and DAMACY discriminant models.

### The effect of pre-processing investigated using Control vs case studies / data

Diagnostic ability is another challenge for which MFC data analysis methods should be used. The multi-set structure allows for pre-processing based on only the control samples to enhance the differences between case/responder and control, which may improve the discrimination and diagnostic ability. In the case of obese versus lean data standard pre-processing already performed very well with 76.6% accuracy, when compared to 76.8% accuracy in the optimal model with control centering and individual scaling. However, the optimal model enhances the response-specific variability in the case samples, and is therefore better able to describe the relationship between the markers measured. This will allow better and more robust interpretation of the data, as shown in the comparison between Fig. 5 and Fig S19 and S20 The high accuracy in standard pre-processing is mainly caused by the high sensitivity, probably because the obese samples had more cells measured and were thus better modeled compared to the control individuals. Also, pre-processing choices could be made that are detrimental to the predictive power, as the worst model based on individual centering and scaling on the whole dataset lead to a prediction accuracy of only 61.4%, which is a decrease of around 15% compared to standard pre-processing. A systematic exploration of all options for multi-set and case-control pre-processing using cross validation and permutation testing is essential to obtain an optimally predictive model^15^. However, an external test set is still required to test the optimal predictive model or should be at least compared with literature and visual inspection is needed for quality control and to interpret the model^6,31^. The current pre-processing setup entails only a limited number of possible permutations, but it needs to be integrated with the other pre-processing steps for MFC, such as transformation and compensation.

## Conclusion

Nowadays most of the widespread applications of MFC involve the measurements of cells from several patients. The same markers are measured across all the patients and this enables the arrangement of MFC data in a multi-set structure. Here we presented how the integration of multi-set structure in the pre-processing and analysis of MFC data led to better interpretation of the analysis methods the results and corrected for challenges occurring in MFC. In fact, the multi-set pre-processing proposed corrects for difference in number of cells measured across all the patients. This difference should be always taken in consideration because it may be detrimental for the interpretation of the findings of the analysis method used, as demonstrated with the simulations proposed.

In addition, the versatility of the pre-processing algorithm allows several different pre-processing strategies. These include solutions to remove unwanted non-biological/technical variation between the samples and strategies to best accommodate the study research questions, *e.g*. discrimination between control and diseased/case group. Control based centering and/or scaling may enhance the effect of the diseased/case group in the model. Individual centering and or scaling may be useful when the model shows an unequal distribution of individual samples, e.g. cells from an individual are only in one part of the t-SNE map or flowSOM tree meaning that the model only describes individual variation instead of between group variation.

The multi-set pre-processing (already present in DAMACY and ECLIPSE algorithms) may be implemented in any multivariate data analysis methods. This may enable outperforming of prediction accuracy and lead to more robust results.

## Supplementary information

Supporting information.

## Data Availability

The data can be downloaded from the website: https://www.ru.nl/science/analyticalchemistry/research/data-analytical-chemistry/

## References

[1] Robinson JP, Roederer M (2015). Flow cytometry strikes gold. Science.

[2] Theunissen P (2017). Standardized flow cytometry for highly sensitive MRD measurements in B-cell acute lymphoblastic leukemia. Blood.

[3] Macaulay IC (2016). Single-Cell RNA-Sequencing Reveals a Continuous Spectrum of Differentiation in Hematopoietic. Cells. Cell Reports.

[4] Tauler R, Maeder M, De Juan A (2009). in Comprehensive Chemometrics: Chemical and Biochemical Data Analysis.

[5] Smilde AK, Westerhuis JA, de Jong S (2003). A framework for sequential multiblock component methods. Journal of Chemometrics: A Journal of the Chemometrics Society.

[6] Saeys Y, Van Gassen S, Lambrecht BN (2016). Computational flow cytometry: helping to make sense of high-dimensional immunology data. Nature Reviews Immunology.

[7] Bruggner RV, Bodenmiller B, Dill DL, Tibshirani RJ, Nolan GP (2014). Automated identification of stratifying signatures in cellular subpopulations. Proceedings of the National Academy of Sciences.

[8] Van Gassen S (2015). FlowSOM: Using self‐organizing maps for visualization and interpretation of cytometry data. Cytometry Part A.

[9] Qiu P (2011). Extracting a cellular hierarchy from high-dimensional cytometry data with SPADE. Nature biotechnology.

[10] Amir E-aD (2013). viSNE enables visualization of high dimensional single-cell data and reveals phenotypic heterogeneity of leukemia. Nature biotechnology.

[11] Tinnevelt GH (2017). Novel data analysis method for multicolour flow cytometry links variability of multiple markers on single cells to a clinical phenotype. Scientific Reports.

[12] Folcarelli R (2018). Automated flow cytometric identification of disease-specific cells by the ECLIPSE algorithm. Scientific reports.

[13] Pillay J (2012). A subset of neutrophils in human systemic inflammation inhibits T cell responses through Mac-1. J Clin Invest.

[14] Wouters K (2017). Circulating classical monocytes are associated with CD11c(+) macrophages in human visceral adipose tissue. Scientific Reports.

[15] Engel J (2013). Breaking with trends in pre-processing?. TrAC Trends in Analytical Chemistry.

[16] Nemes E (2015). Differential leukocyte counting and immunophenotyping in cryopreserved *ex vivo* whole blood. Cytometry Part A.

[17] de Ruiter K (2018). A field-applicable method for flow cytometric analysis of granulocyte activation: Cryopreservation of fixed granulocytes. Cytometry Part A.

[18] Johnson NL (1949). Systems of Frequency Curves Generated by Methods of Translation. Biometrika..

[19] Roederer M (2001). Spectral compensation for flow cytometry: visualization artifacts, limitations, and caveats. Cytometry.

[20] Finak G, Perez JM, Weng A, Gottardo R (2010). Optimizing transformations for automated, high throughput analysis of flow cytometry data. BMC bioinformatics.

[21] Bro R, Smilde AK (2003). Centering and scaling in component analysis. Journal of Chemometrics.

[22] Joliffe, I. T. Principal Component Analysis, Second Edition. Vol. 98 (Springer, 1986).

[23] Lugli E, Roederer M, Cossarizza A (2010). Data analysis in flow cytometry: the future just started. Cytometry. Part A: the journal of the International Society for Analytical Cytology.

[24] Timmerman ME, Kiers HAL (2003). Four simultaneous component models for the analysis of multivariate time series from more than one subject to model intraindividual and interindividual differences. Psychometrika.

[25] Westerhuis, J. A., Kourti, T. & MacGregor, J. F. Analysis of multiblock and hierarchical PCA and PLS models. *Journal of Chemometrics* **12**, 301-321, 10.1002/(SICI)1099-128X(199809/10)12:5<301::AID-CEM515>3.0.CO;2-S (1998).

[26] Gower, J. C., Lubbe, S. & Le Roux, N. Understanding Biplots. (Wiley, 2011).

[27] Pillay J (2010). Functional heterogeneity and differential priming of circulating neutrophils in human experimental endotoxemia. Journal of leukocyte biology.

[28] Hahne F (2010). Per‐channel basis normalization methods for flow cytometry data. Cytometry Part A: The Journal of the International Society for Advancement of Cytometry.

[29] Vesanto, J., Himberg, J., Alhoniemi, E. & Parhankangas, J. Self-organizing map in Matlab: the SOM Toolbox. Proceedings of the Matlab DSP conference **99** (1999).

[30] Trygg J, Wold S (2002). Orthogonal projections to latent structures (O-PLS). Journal of Chemometrics.

[31] Szymańska, E., Saccenti, E., Smilde, A. & Westerhuis, J. Double-check: validation of diagnostic statistics for PLS-DA models in metabolomics studies. *Metabolomics* **8**, 3–16. 10.1007/s11306-011-0330-3 PMC3337399 22593721

