# Supplementary material for: Multi-set Pre-processing of Multicolor Flow Cytometry Data
Source: Sci Rep. 2020 Jun 16;10:9716. doi: 10.1038/s41598-020-66195-3 (PMC7297713; doi:10.1038/s41598-020-66195-3)
Supplement: Supplementary file 1 — Supporting information. [file 41598_2020_66195_MOESM1_ESM.docx]

**Multi-set pre-processing of Multicolor Flow Cytometry Data**

**Rita Folcarelli^a*^, Gerjen H. Tinnevelt^a,b*^, Bart Hilvering^c^, Kristiaan Wouters^d^, Selma van Staveren^b,c^, Geert J. Postma^a^, Nienke Vrisekoop^c^, Lutgarde M. C. Buydens^a^, Leo Koenderman^c^, Jeroen J. Jansen^a^**

*^a^Radboud University, Institute for Molecules and Materials, Analytical Chemistry, P.O. Box 9010, 6500 GL Nijmegen, The Netherlands
^b^TI-COAST, Science Park 904, 1098 XH Amsterdam, The Netherlands
^c^Department of Respiratory Medicine laboratory of translational immunology, University Medical Center Utrecht, Heidelberglaan 100, 3584CX, Utrecht, The Netherlands*

*^d^Department of Internal Medicine, Laboratory of Metabolism and Vascular Medicine, P.O. Box 616 (UNS50/14), 6200 MD Maastricht, The Netherlands*

**Authors contributed equally to the work*

Contents

[Supplementary material I 3](#_Toc528743901)

[Simulated Data 3](#_Toc528743902)

[Supplementary Material II 5](#_Toc528743903)

[LPS data 5](#_Toc528743904)

[Supplementary Material III 10](#_Toc528743905)

# Supplementary material I

## Simulated Data


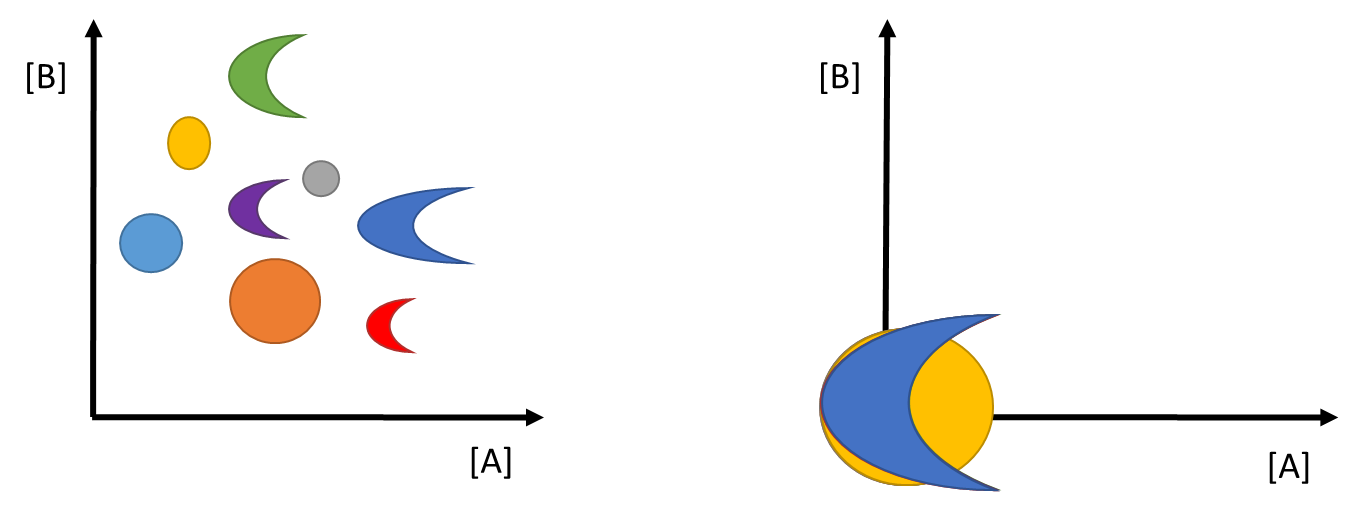


Scheme S1: Schematic representation of large technical variability between samples and how shape may still be important after removing the individual mean and standard deviation of marker A and B. The different colors show the different samples and the shape show the group, circle or moon. The left panel is before pre-processing and the right panel is after individual centering and scaling. In the right panel all samples have the same mean value and have the same standard deviation, however, you still see the difference between circle and moon.

# Effect of sample size, illustrated with simulated data with increasing complexity

### Simulated data

Simulated dataset A was constructed to illustrate the effect of the different pre-processing strategies. It consisted of two normal distributions, representing different cell populations (Fig S1). A ‘control’ population (red rounds) was drawn from a normal distribution with mean μ_control_ = 4,0 and standard deviation σ_control_= 0.5, 2 and a ‘responder’ population (blue crosses) from a normal distribution with mean μ_response_ = 4,4 and standard deviation σ_response_ = 2, 0.5. The responder population shows a different shape and it is shifted compared to the control population.

Figure S 1: 2D scatter plot of simulated data **A**. A ‘control’ population is depicted in red, while ‘responder’ population is visualized in blue. The responder population shows a different shape and it is shifted compared to the control population

Simulated datasets B, C and D were created to illustrate the influence of different sample sizes in the estimation of the mean and how this would affect representation of the pre-processed data. All the simulations were characterized by three samples having increasingly complexity in number of variables and cell populations. In simulated dataset B, the three samples comprised two variables and were characterized by a single population. The event size of the samples was initially the same: 10.000 events each. The event size of one sample was then increased to 100.000, 250.000, 500.000 events, while maintaining the size of both other samples invariant. In dataset C another combination of three samples was successively created by adding a third variable and a mutual exclusive positive expression of only one of the three markers on the different samples. A more complex dataset D was finally introduced to mimic a more realistic immunological scenario with 6 variables and presence of small subsets in some of the samples. For the latter two simulations, the event size of one sample was enlarged to 500.000 compared to the other two samples that maintained the same size of 10.000 events. The effect of pre-processing on such constructed higher dimensional data was investigating by applying the dimensionality reduction method Simultaneous Component Analysis (SCA). Two-dimensional synthetic dataset B is constructed to highlight the effect of changes in the number of events (cells) between samples on the calculated mean.

The data comprises three samples coded in different colours. Each sample is represented by a single cell population, of which variables expression can be observed in the univariate histograms shown in Fig S2. Fig S3 shows the change in value of the overall mean, calculated according to Eq. 2b, when the size of the green sample progressively increases. As the ratio of the event size between the green and the other two groups increases, the mean shifts towards the centre of mass of the most populated sample.


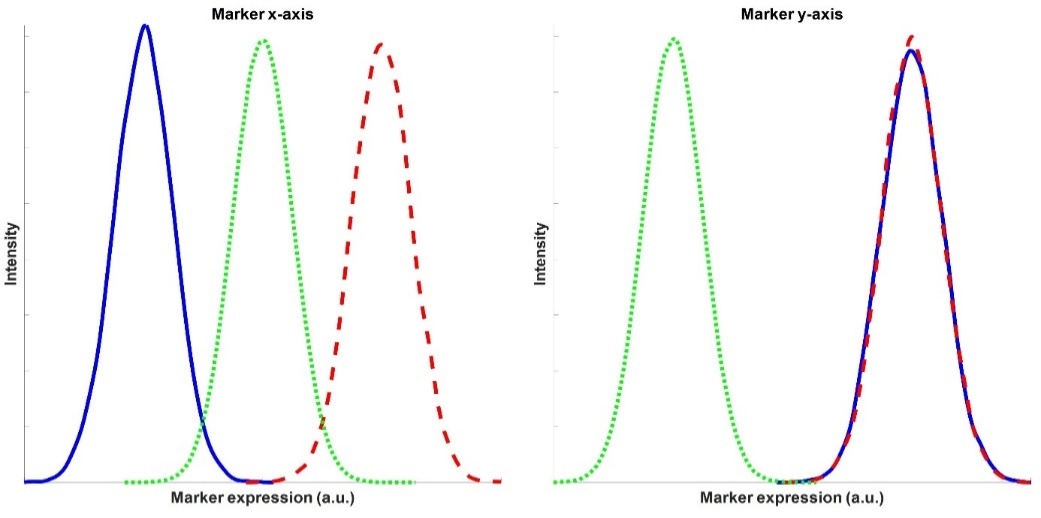


Figure S 2: Histograms showing the expression of the two variables included in the 2-dimensional synthetic data. The three populations blue, red and green are displayed with corresponding colors and different line style.


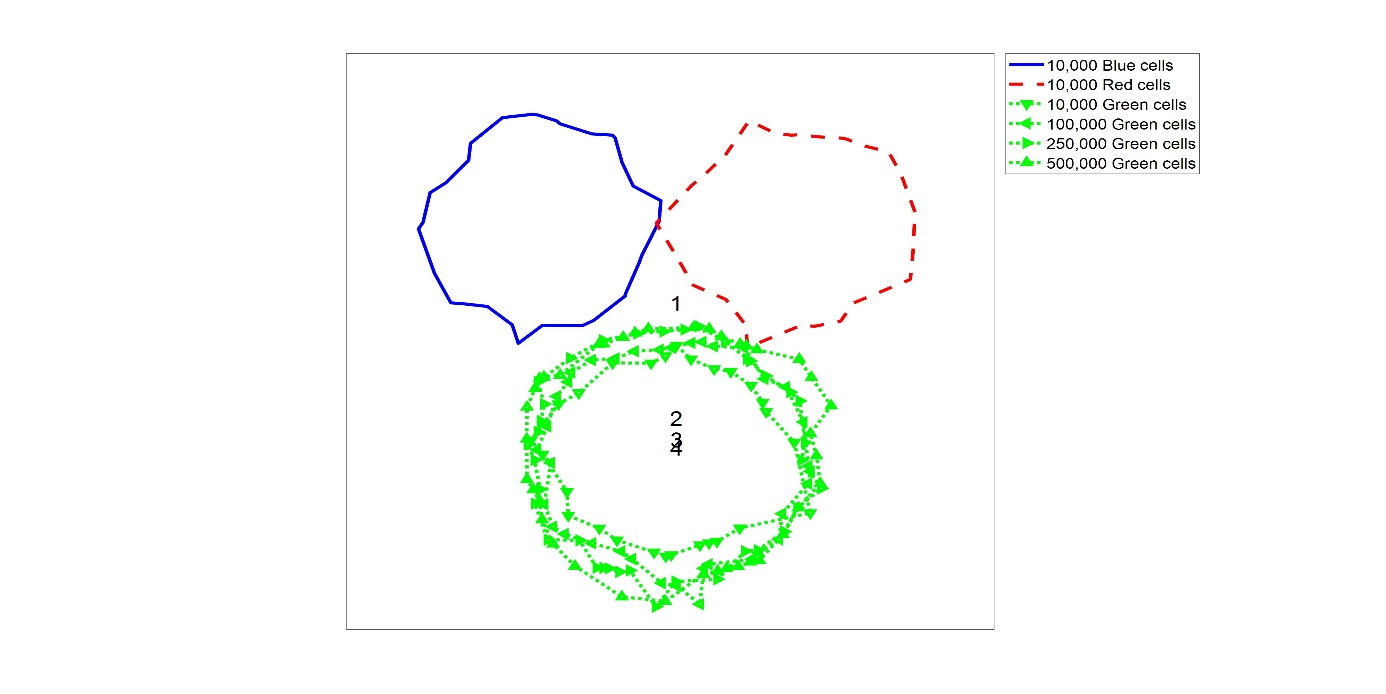


Figure S 3: Contour representation of simulated data consisting of three samples (blue, red, green), characterized each by a single population. The numbers represent the position of the mean calculated on the merged data, after increasing the size of the green sample: 1. samples size is the same, 10.000; 2. sample size of the green sample increased to 100.000; 3. sample size of the green sample increased to 250.000; 4. sample size of the green sample increased to 500.000.

This shift of the mean may change interpretation of the data, as it greatly affects the results of multivariate analysis methods that combine information of the three surface markers. We illustrate the effects of sample sizes on pre-processing of higher dimensional simulated dataset by applying the widely used data reduction and visualization technique Simultaneous Component Analysis (SCA).

Simulated dataset C has three samples with a single cell population. The histograms of the three markers expression level are shown in the Online Supplementary Material I (Fig S4).
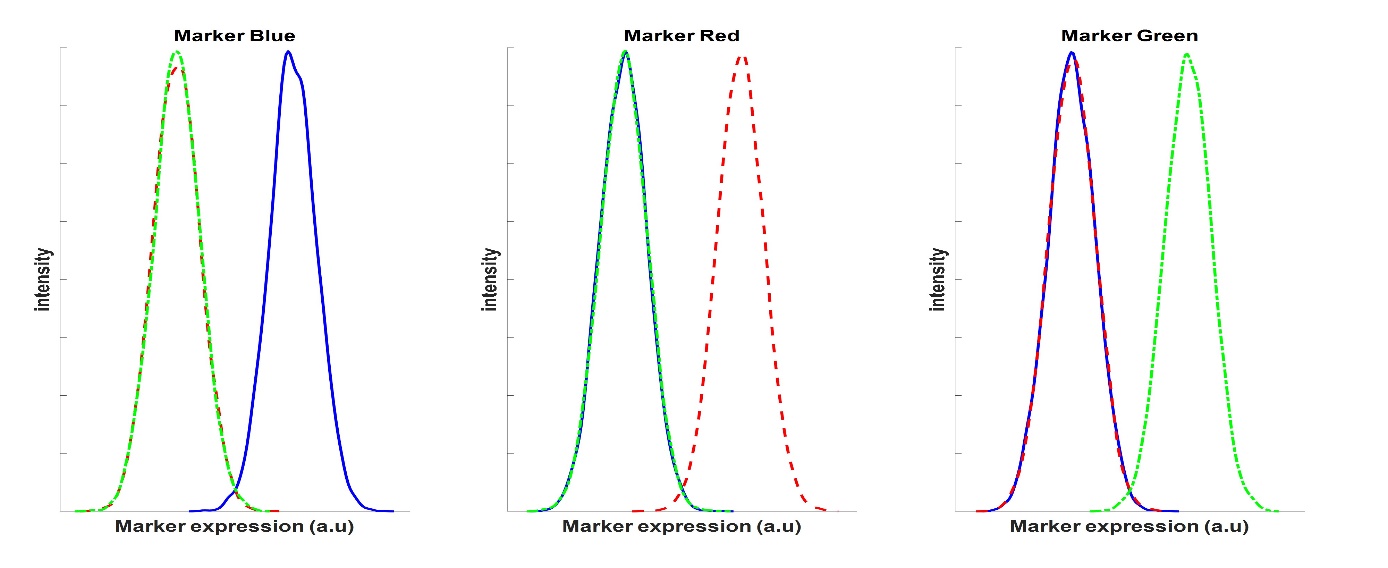


Figure S 4: Histograms shown the marker expression level of the three markers created for the synthetic data consisting of three single-population groups blue, red and green. Each group is represented by the corresponding color and different line style and it is characterized by the exclusive (positive) expression of the marker of the corresponding color.

The three samples are characterized by the mutual exclusive positive expression of only one of the three markers: the blue group is positive for Blue, the red group is positive for Red and the green group is positive for Green. Similar to the previous simulation, we increased the original size of the green sample of factor 50 compared to the size of the blue and red samples. The resulting simulated dataset with different sample size was pre-processed using conventional pre-processing (Eq. 2-3) and multi-set pre-processing over the whole dataset (Eq. 4 and 7). SCA models were built on the resulting different simulated subsets and the effect of the analyses on the diverse mean-centered (and block-scaled) data is shown in Fig S5.

**
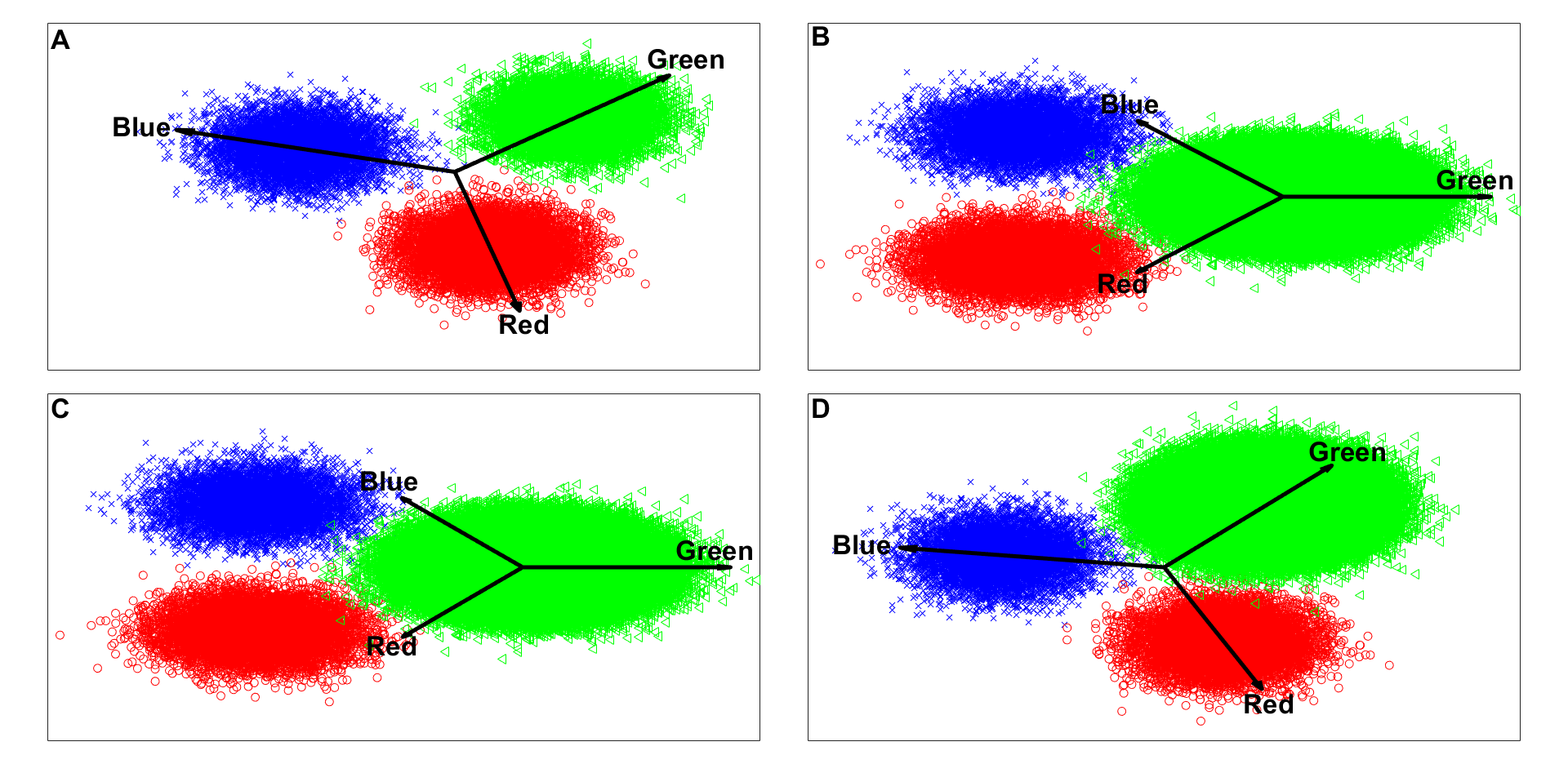
**

Figure S 5: Biplot of SCA model. A single population for each sample, the arrows show loadings. A) Standard SCA model with same sample size of 10.000; B) Standard SCA model and sample size of the green triangles increased to 500.000; C) Block scaled SCA model and sample size of the green triangles increased to 500.000; D) Multi-set pre-processing over the whole dataset (Eq. 4 and 7) and block scaled SCA model and sample size of the green triangles increased to 500.000.

When the sample sizes is equal among the three samples (Panel A), the origin of the loadings is in the middle of the data, suggesting that the mean properly represents the average marker expression among the three samples. Moreover, it can be observed that the loadings point toward the correct cell populations on which each loading is exclusively expressed. In Panel B, the size of the green group was increased 50 fold and when SCA is applied to the conventionally mean-centered data, the Green loading is large and positive (points to the right) in the first principal component while the Blue/Red loading is smaller and negative (points to the left). Additionally, as effect of conventional centering the origin is moved in the direction of the green sample and this would indicate that it has an average expression of all markers. In reality, the green sample is low in Red and Blue, and high only in Green, as shown in the single marker expression histograms (Fig S4, Online Supplementary Material I). Block scaling (Equation 9a) applied before the SCA model does not overcome this misinterpretation, see Panel C. In fact, only when combining block scaling with multi-set pre-processing (Panel D), then the SCA model is similar to the one shown in Panel A, which means the analysis is not influenced anymore by the different numbers of cells within the samples.

In simulated dataset D three additional variables (GreenBlue, GreenRed and AntiGreen) are added (Figure S6), where the red and the blue samples have an extra small cell population positive for Red and Blue variable, respectively. All three samples have a similar cell population which is positive for variables Green, GreenRed, GreenBlue and negative for variables Blue, Red and Antigreen.


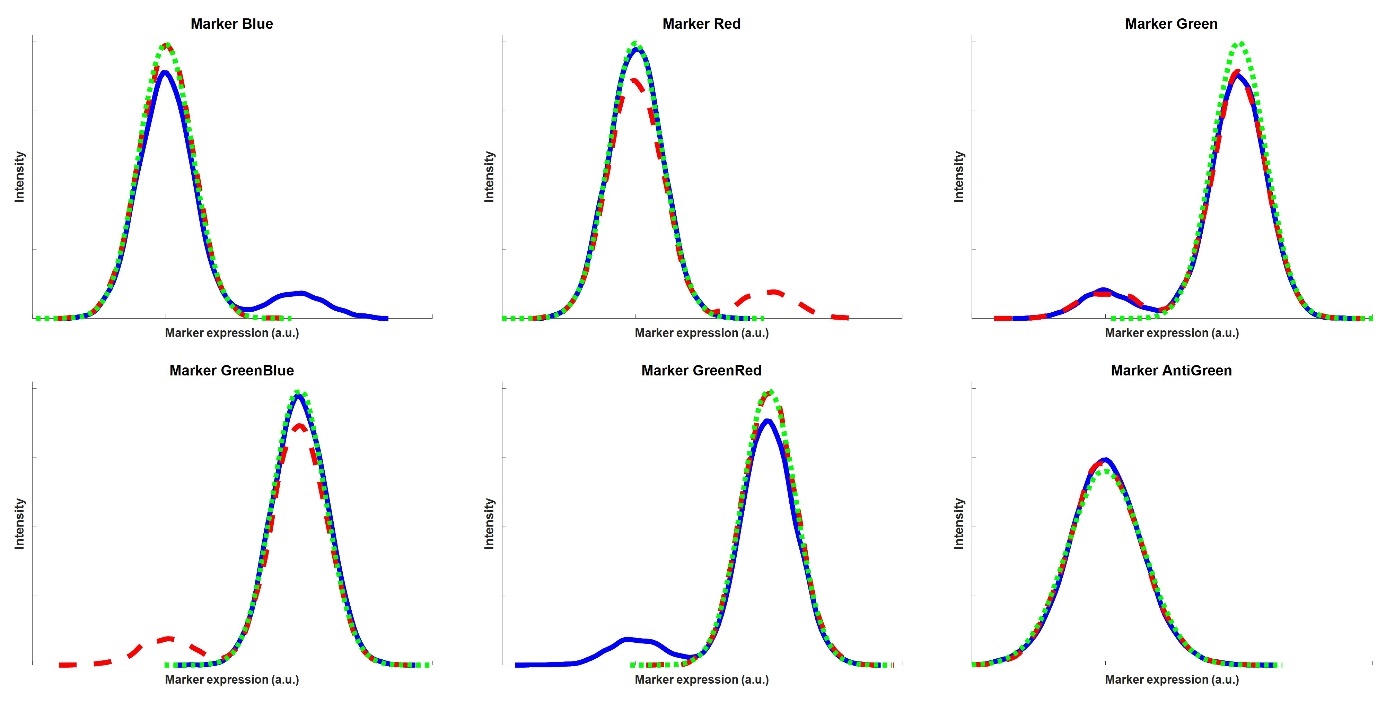


Figure S 6: Histograms shown the marker expression level of the six markers created for the synthetic data consisting of three samples colored blue, red and green. All three samples have a common cell population with high expression for Green, GreenRed, Greenblue and low for Blue and Red. The blue and red sample have an extra small population high for Blue and Red respectively and low for GreenRed and Greenblue respectively and both are low for Green. Variable AntiGreen is negative correlated to Green.

Again, the three MFC samples have the same size of 10.000 cells, here divided in 1000 cells and 9000 within the small and the big population, respectively, for both blue and red sample. The green sample was then 50 fold increased in size in the same manner as previously. SCA analyses are performed and shown in Fig S7. When the three samples have the same number of cells measured (Panel A), the origin of the SCA model is in the similar population, overlapping as well with the major population of blue and red.

**
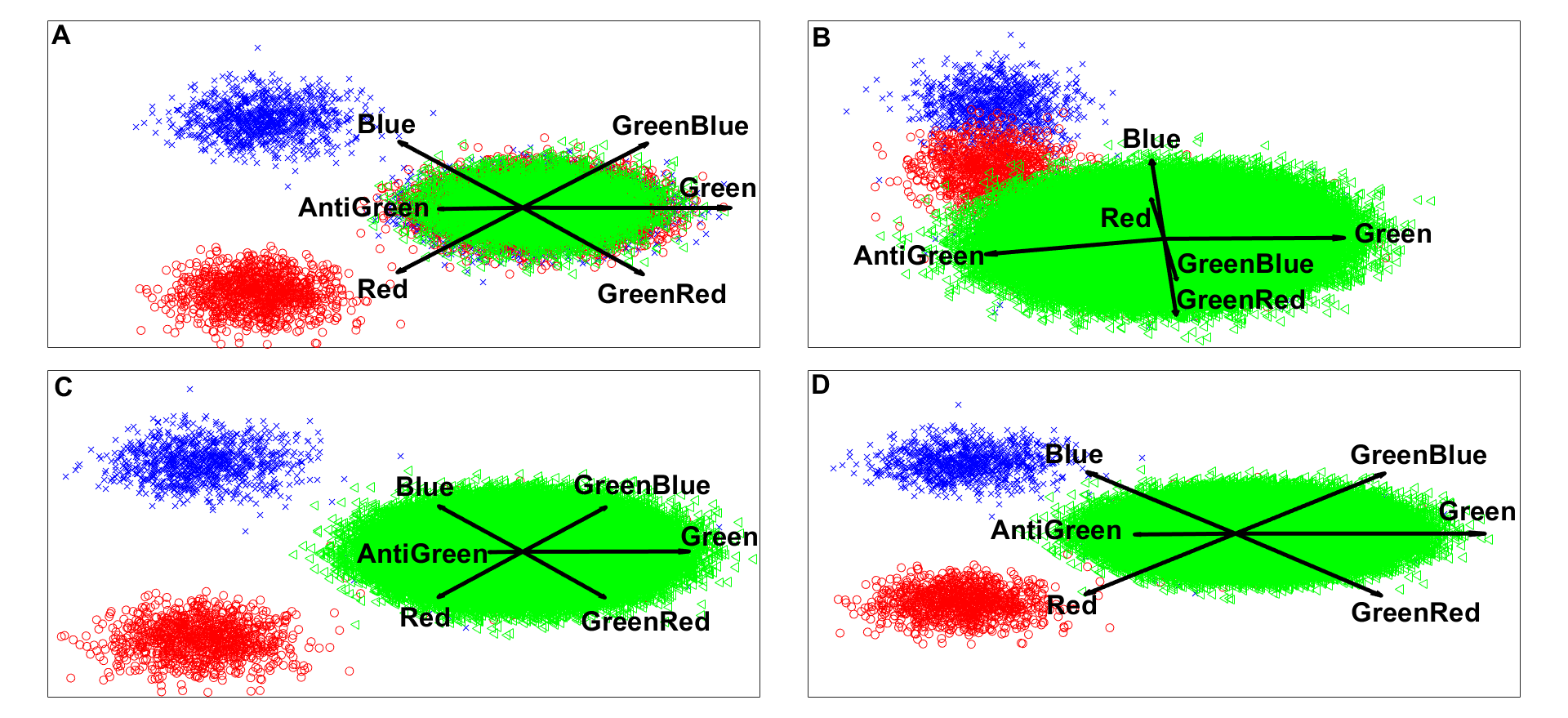
**

Figure S 7: Biplot of SCA model. A) Standard SCA model with same sample size of 10.000 for all 3 populations; Blue crosses have both 1000 cells in the blue population and 9.000 in green population, red circles have both 1000 cells in the red population and 9000 in green population, green triangles have 10.000 cells in the green population. B-D) sample size of the green triangles increased to 500,000; B) Standard SCA model C) Block scaled SCA model D) Multi-set pre-processing and block scaled SCA model.

In panel B the green sample has increased in size and the resulting data are conventionally mean-centered. When SCA is applied, the first two principal component to mainly describe the variance within the green group. The extra red and extra blue population overlap partly and they are very close to the green populations. The loadings do not correctly describe the variability of these two small populations. In the third analysis the block scaling helps in equally weighting the samples in the built SCA model (Panel C). The extra red and extra blue population are now well described and the loadings seem to be correctly oriented. However, the loading of AntiGreen variable is very small, meaning that this marker does not contribute much to the model and thus it is not a descriptor for the populations. A more relevant representation of the marker expressions, that contains the systematic variation introduced into the simulation, is obtained only after multi-set pre-processing over the whole dataset and block scaling (Panel D). The resulting SCA model is similar to the model in Panel A. Multi-set pre-processing combined with block scaling is essential to represent the different cell populations accurately and to reflect the original marker expression.

# Supplementary Material II

## LPS data

**
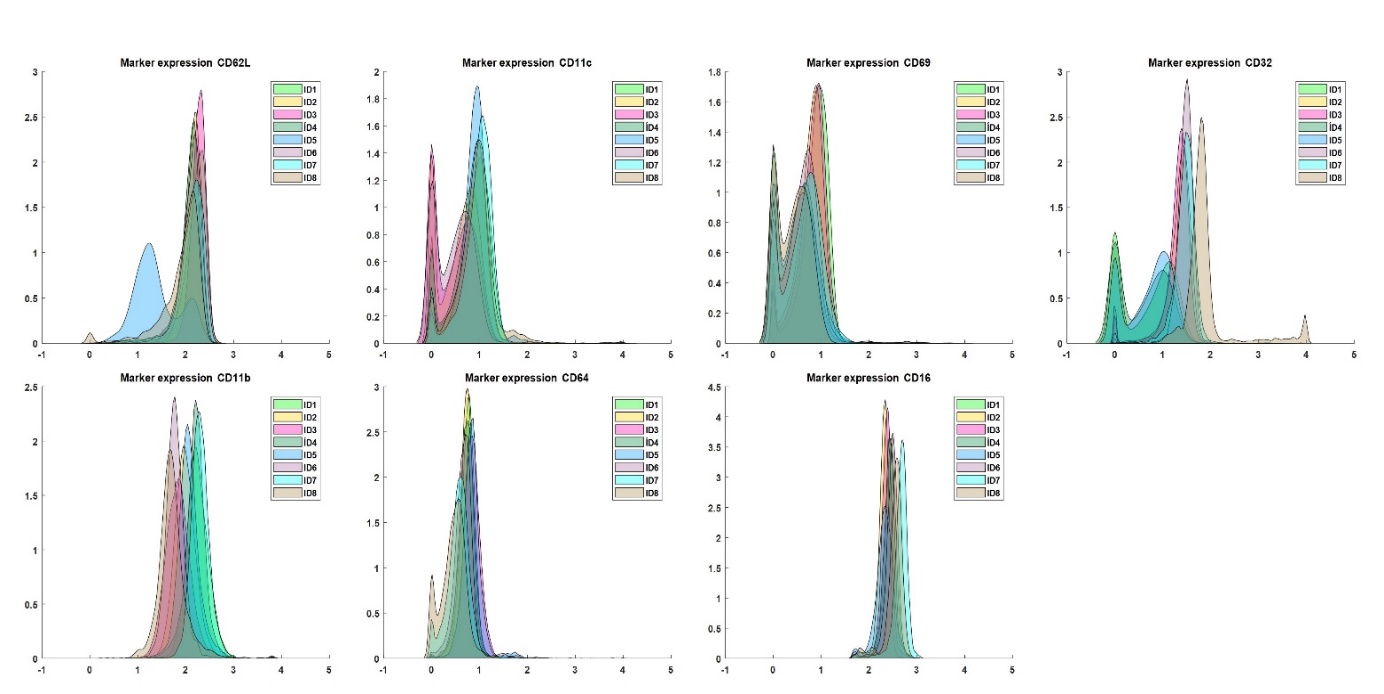
**


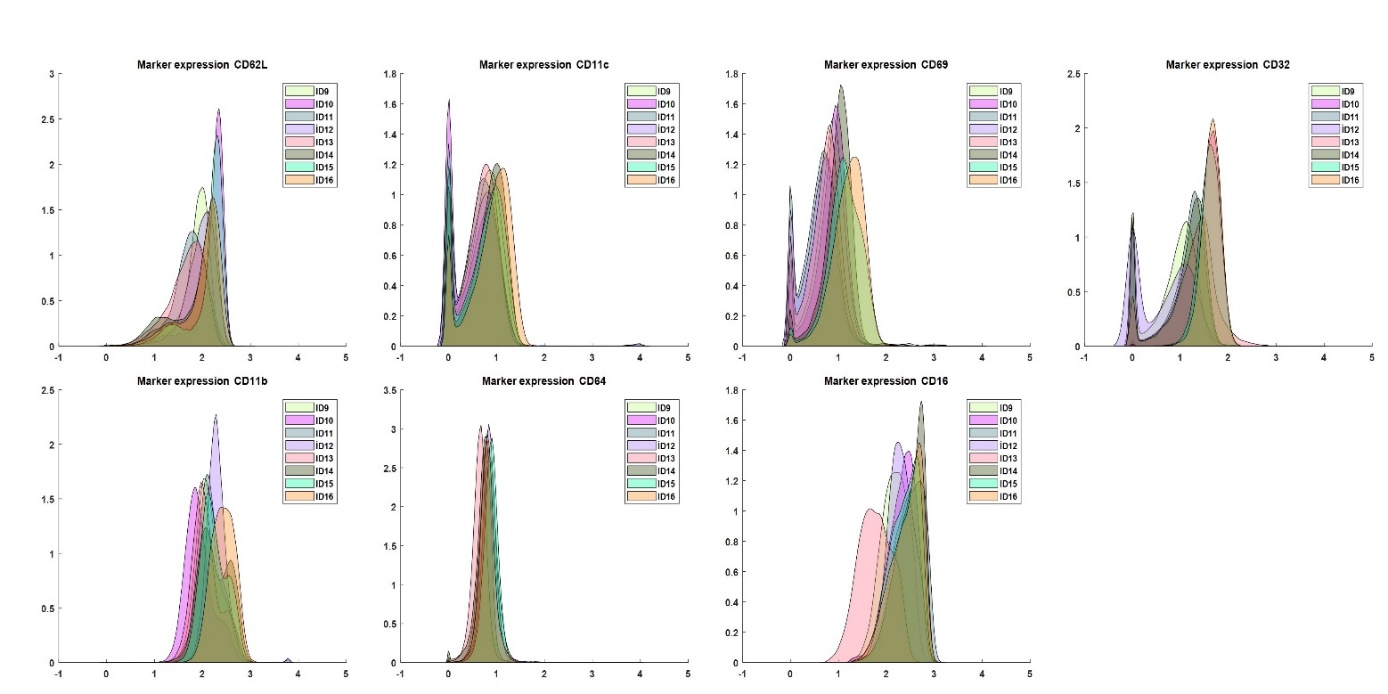


Figure S 8: Panel A: Histograms of the original single marker expression of control individuals. Different individuals are displayed in different colours; Panel B: Histograms of the original single marker expression of responder individuals. Different responder individuals are displayed in different colors. Marker CD16 shows a broader distribution in responders compared to controls.


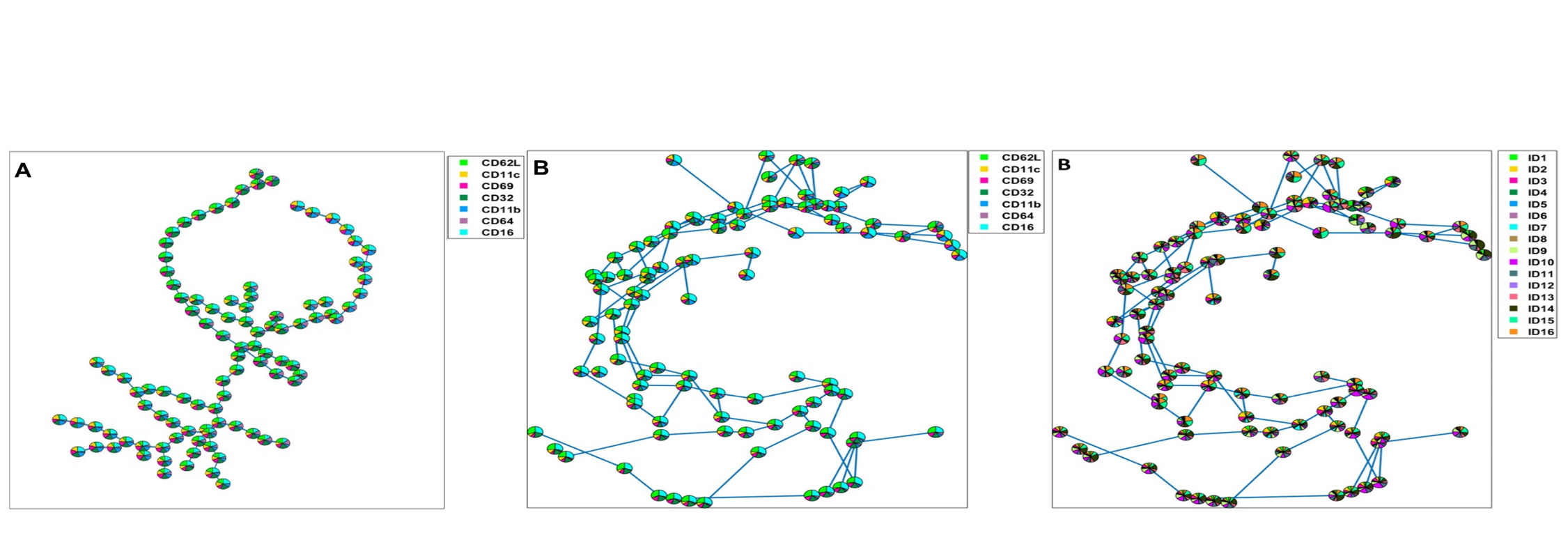


Figure S 9: SOM analysis results. Nodes of the SOM trees are colored according to the mean intensities of the markers for all the cells assigned to each node. Panel **A**: SOM tree results obtained for the conventionally pre-processed LPS dataset, consisting of centering and scaling by using mean and standard deviation calculated over the all the samples; Panel **B**: SOM tree results obtained for the multi-set pre-processed LPS dataset, consisting of centering and scaling over the control individuals.


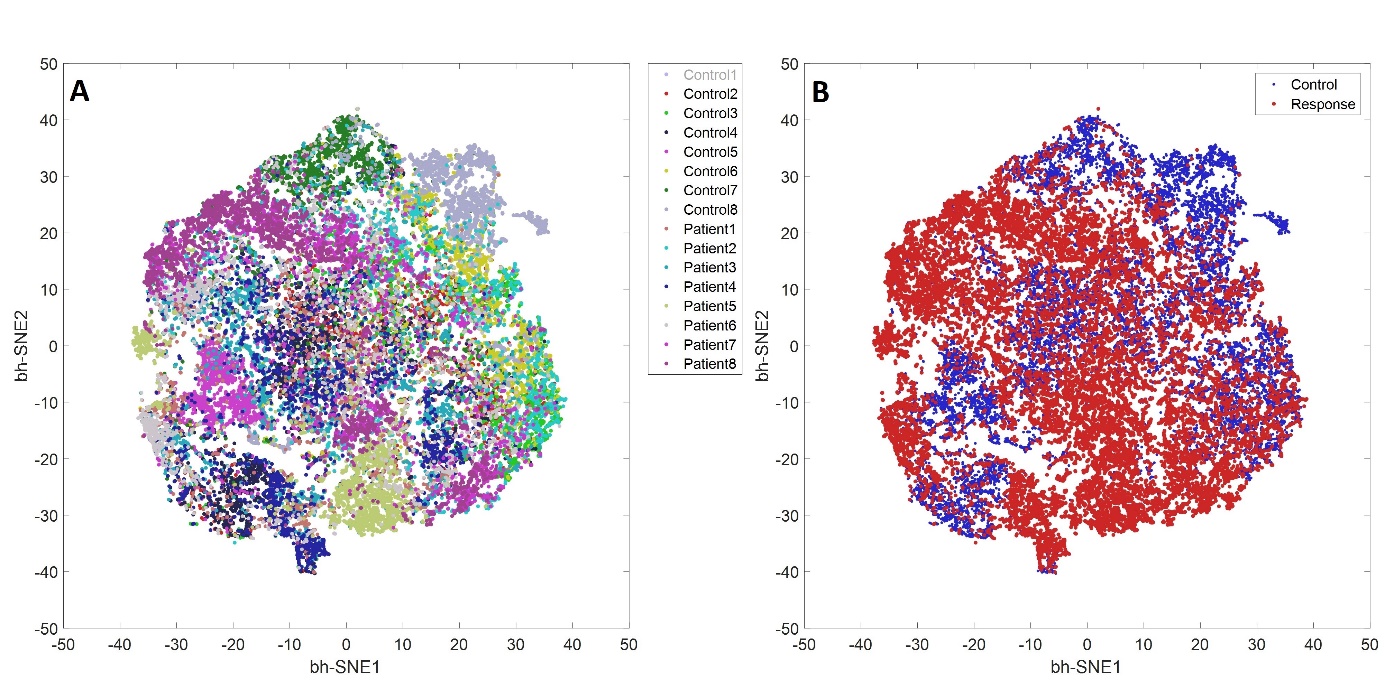


Figure S 10: viSNE analysis performed on the conventionally pre-processed LPS data, consisting of centering and scaling by using mean and standard deviation calculated over the all the samples. Panel A: cells in the viSNE map are colored per different sample; Panel B: cells in the viSNE map are colored based on control (blue) and responder (red) group.


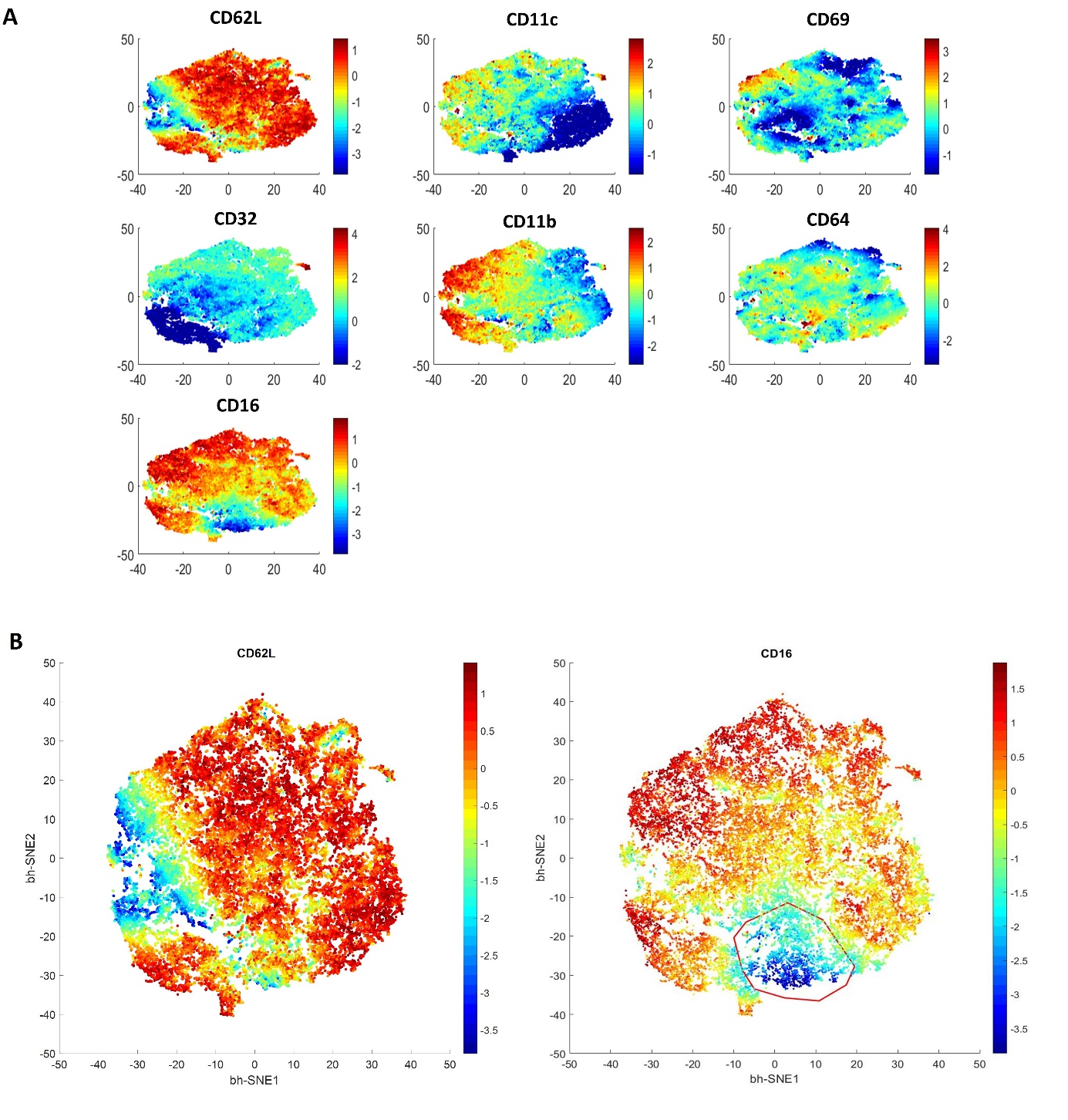


Figure S 11: viSNE analysis performed on the conventionally pre-processed LPS data, consisting of centering and scaling by using mean and standard deviation calculated over the all the samples. Panel A: Cells are colored based on expression of the single 7 surface markers. Panel B: CD62L and CD16 expression plots are zoomed; the region in the viSNE which may be assigned to premature neutrophils (CD16-CD62L+) is gated for further inspection.


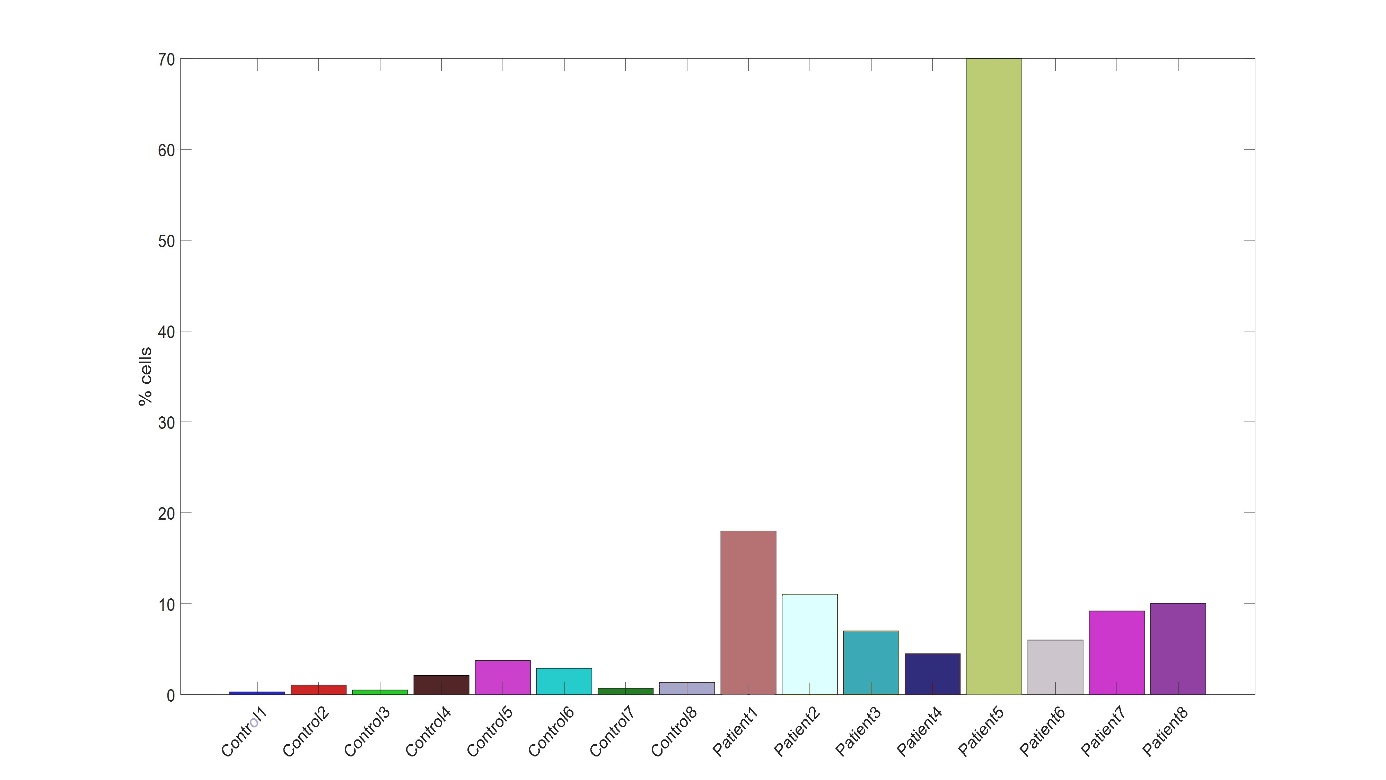


Figure S 12: Barplot of the amount of cells per each individual present in the gated region which may be identified as premature neutrophils of the viSNE map. Cells contained in that region are mainly from the responder individual Patient #5.


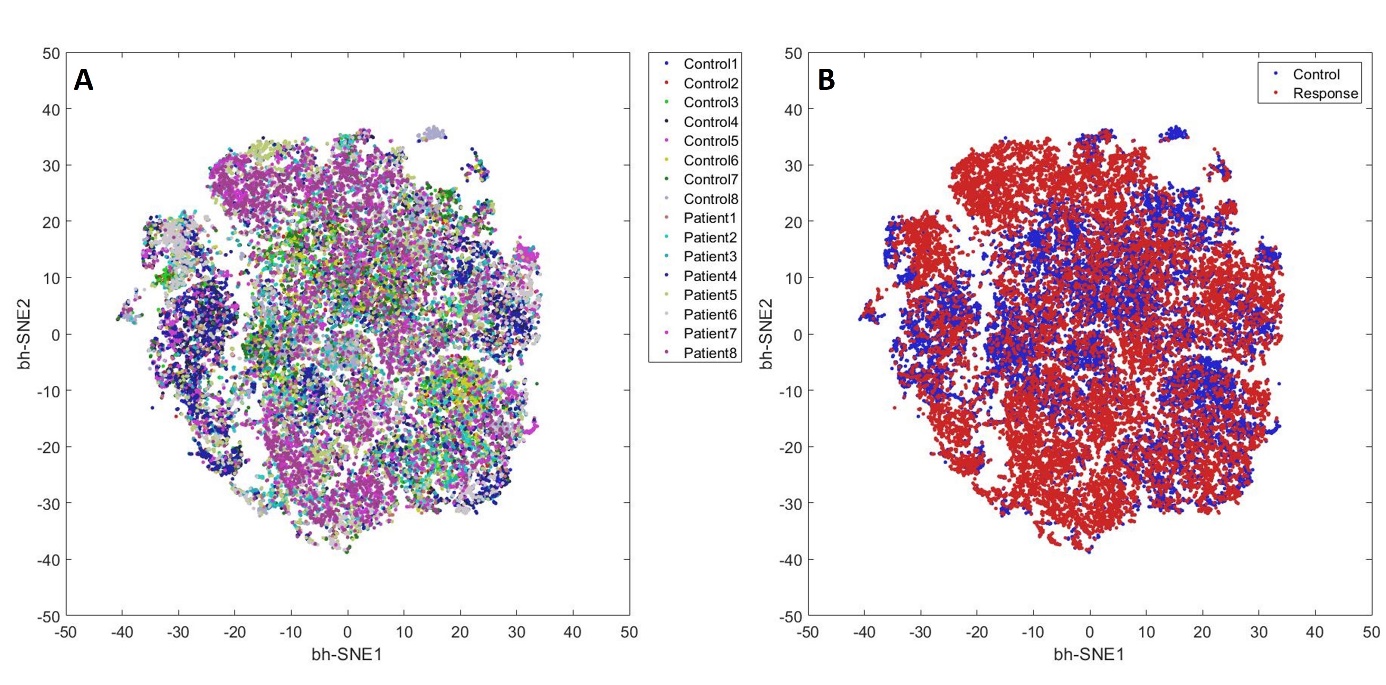


Figure S 13: viSNE analysis performed on the conventionally pre-processed LPS data, consisting of centering and scaling over the control individuals. Panel A: cells in the viSNE map are colored per different individual; Panel B: cells in the viSNE map are colored based on control (blue) and responder (red) group.


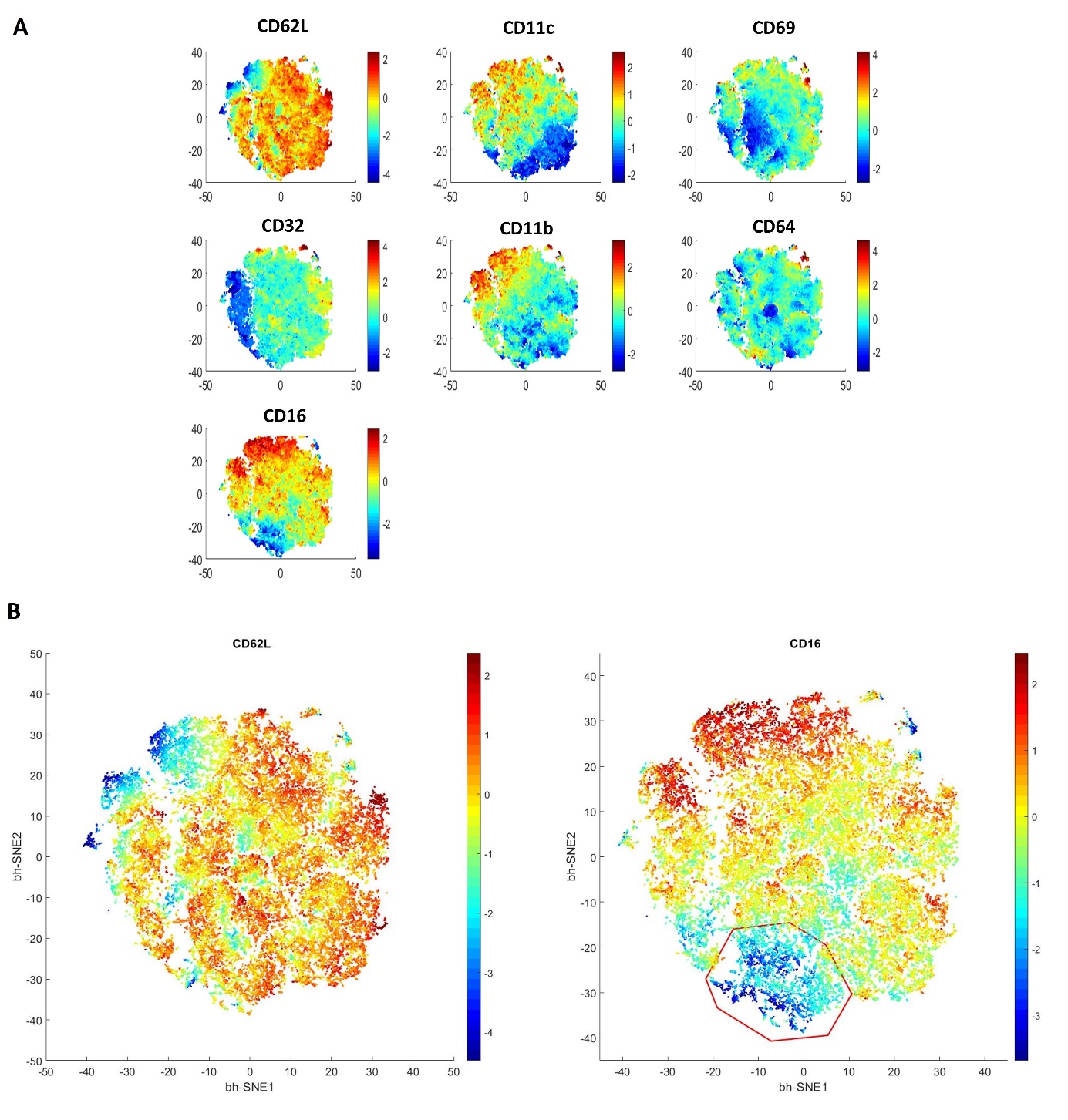


Figure S 14: viSNE analysis performed on the multi-set pre-processed LPS data, consisting of centering and scaling over the control individuals. Panel A: Cells are colored based on expression of the single 7 surface markers. Panel B: CD62L and CD16 expression plots are zoomed; the region in the viSNE which may be assigned to premature neutrophils (CD16-CD62L+) is gated for further inspection.


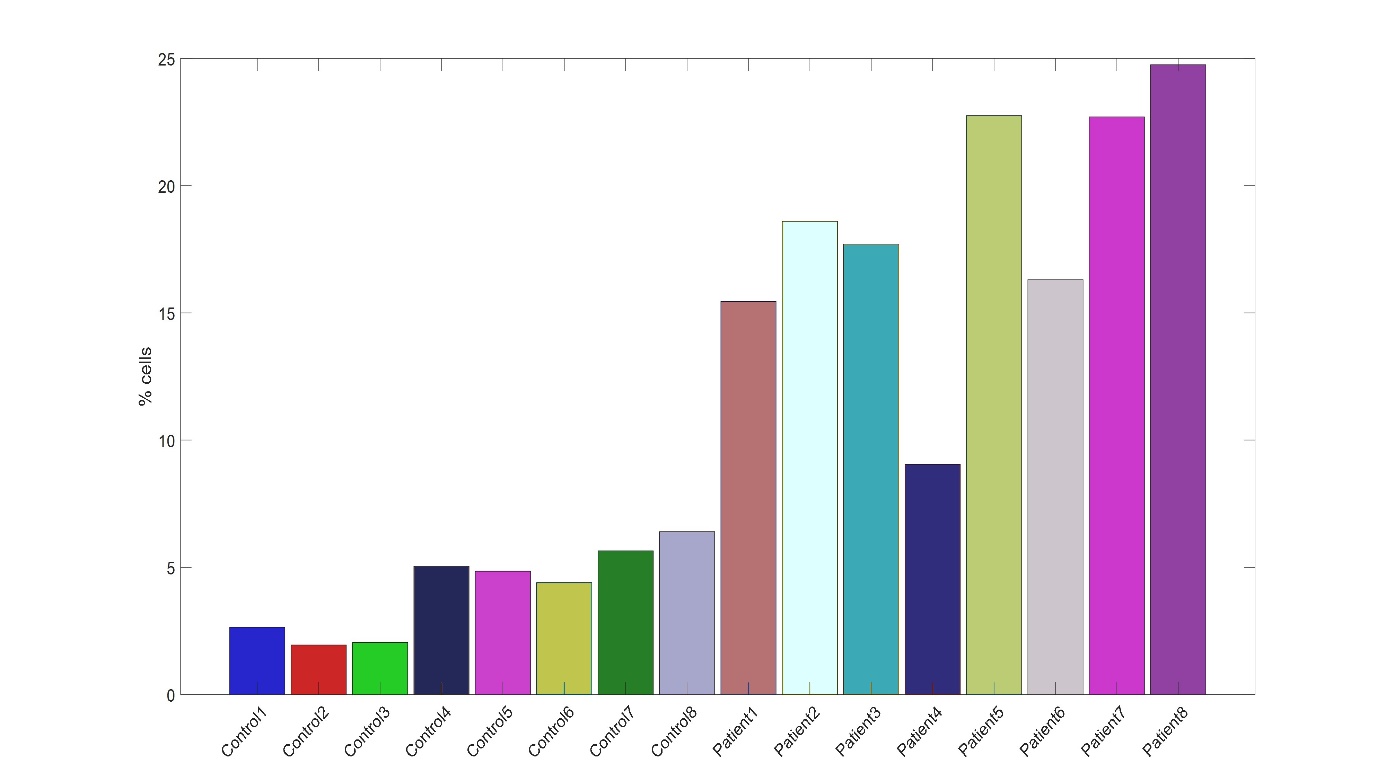


Figure S 15: Barplot of the amount of cells per each individual present in the gated region CD16-CD62L+ which may be identified as premature neutrophils of the viSNE map. Cells contained in that region from all the responder individuals; also control individuals have small percentage of cells in the gated region.


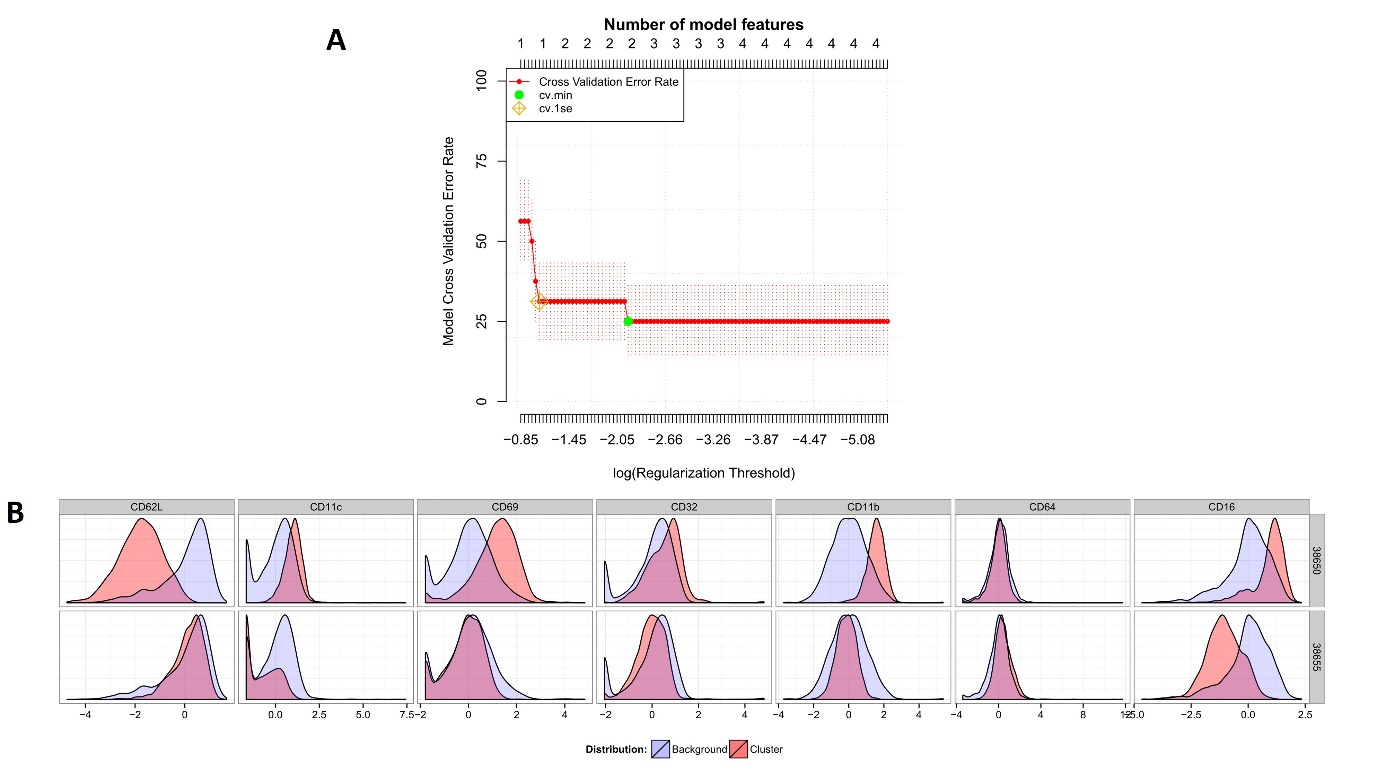


Figure S 16: Panel A: The Figure shows the Model Cross-validation Error Rate vs the log(Regularization Threshold) for the classification models constructed on the standard pre-processed LPS dataset. The green circle (cv.min) points out the model with the smallest number of features necessary to obtain the lowest cross-validation error; while the orange diamond (cv_1se) indicates the model with the smallest number of features associated to cross-validation error 1 std higher than the minimum error. The model with cv.min is chosen by the Citrus analysis and this corresponds to an error rate of around 25%; Panel B: The histograms show the phenotype of the cells belonging to the cluster (red) selected by the cross-validated model. The background histograms (blue) show the rest of the data, not included in the cluster. Cells from cluster 38650 are characterized by the distinctive phenotype CD16+CD11b+CD69+CD11c+CD62L-; cells from cluster 38655 are CD16-CD62L+CD64+. Both clusters are more abundant in the responder group.


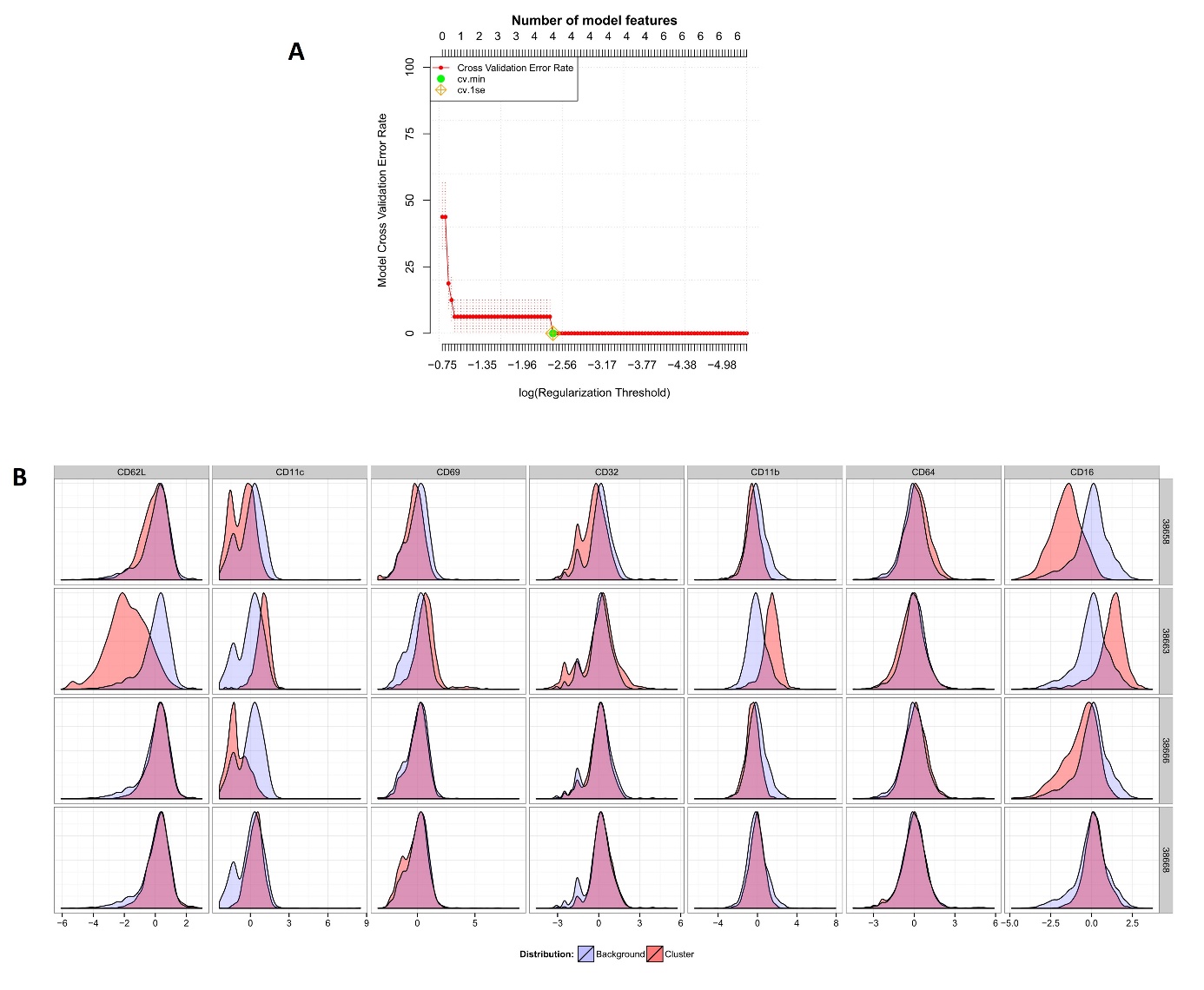


Figure S 17: Panel A: Cross-validation error rate plot for the analysis on the multiset pre-processed LPS dataset. Model Cross Validation Error Rate equals to 0 is obtained with 4 stratifying features. Panel B: Histograms show the phenotypes of the four cluster identified by the cross-validated classification model. The first three clusters are more abundant in the responder group: cluster 38658 and 38666 present phenotypes that might be assigned to pre-mature neutrophils, with CD16-CD62L+andmid; cluster 38663 might be identified as mature neutrophils with CD16+CD11b+CD62L-. Cluster 38668, more abundant in the control group, can be associated to mature neutrophils.

# Supplementary Material III

Supplementary Table 1: Prediction performance of the LPS data with DAMACY using different pre-processing strategies. The optimal strategy chosen is highlighted in bold. All models below a 94% accuracy have a permutation p-value of higher than 0.05 and are not significant.

| **Centering** | **Scaling** | **Accuracy** | **Sensitivity** | **Specificity** | **AUC** | **Mean #OLV** | **p-value** |
| --- | --- | --- | --- | --- | --- | --- | --- |
| Standard | Standard | 75% | 88% | 63% | .86 | 4 | <720/1000 |
| Whole | Whole | 81% | 100% | 63% | 1 | 6 | <356/1000 |
| Control | Whole | 88% | 100% | 75% | .94 | 6 | <123/1000 |
| **Individual** | **Whole** | **100%** | **100%** | **100%** | **1** | **1** | **<4/1000** |
| Whole | Control | 88% | 100% | 75% | .84 | 5 | <123/1000 |
| Control | Control | 75% | 88% | 63% | .83 | 6 | <720/1000 |
| Individual | Control | 94% | 100% | 88% | 1 | 1 | <21/1000 |
| Whole | Individual | 50% | 63% | 38% | .58 | 6 | 1 |
| Control | Individual | 25% | 38% | 13% | .39 | 6 | 1 |
| **Individual** | **Individual** | **100%** | **100%** | **100%** | **1** | **0** | **<4/1000** |

Supplementary Table 2: Prediction performance of the Obesity versus lean data with DAMACY using different pre-processing strategies. The optimal strategy chosen is highlighted in bold. All models below a 74% accuracy have a permutation p-value of higher than 0.05 and are not significant.

| **Centering** | **Scaling** | **Accuracy** | **Sensitivity** | **Specificity** | **AUC** | **Mean #OLV** | **p-value** |
| --- | --- | --- | --- | --- | --- | --- | --- |
| Standard | Standard | 76.6% | 81.9% | 70.0% | .860 | 7 | 28/1000 |
| Whole | Whole | 75.9% | 76.8% | 74.5% | .826 | 4 | 32/1000 |
| Control | Whole | 75.6% | 78.6% | 72.2% | .820 | 5 | 35/1000 |
| Individual | Whole | 61.4% | 70.1% | 50.4% | .637 | 4 | 507/1000 |
| Whole | Control | 75.6% | 78.5% | 72.0% | .835 | 5 | 35/1000 |
| Control | Control | 76.1% | 78.5% | 73.1% | .847 | 6 | 30/1000 |
| Individual | Control | 62.1% | 71.0% | 51.1% | .636 | 3 | 475/1000 |
| Whole | Individual | 76.7% | 78.1% | 74.9% | .831 | 7 | 27/1000 |
| **Control** | **Individual** | **76.8%** | **78.4%** | **74.8%** | **.845** | **7** | **27/1000** |
| Individual | Individual | 62.7% | 71.2% | 52.2% | .646 | 4 | 440/1000 |

Figure S 18: Receiver Operating Characteristic (ROC) curve of the prediction performance on the discrimination of lean versus obese individuals with DAMACY using different pre-processing strategies

Figure S 19: DAMACY model of obese versus lean data with conventional pre-processing. The left panel shows the average prediction score of the OPLS-DA model of controls as red rounds and asthma individuals as blue crosses. The right panel shows negative weights as red and positive weights as blue. The loadings of the Base model are plotted on top as black vectors and indicate how each surface marker contributes to the cell variability in a specific direction within the model.

Figure S 20: DAMACY model of obese versus lean data with worst pre-processing, centering per individual and scaling based on the whole dataset. The left panel shows the average prediction score of the OPLS-DA model of controls as red rounds and asthma individuals as blue crosses. The right panel shows negative weights as red and positive weights as blue. The loadings of the Base model are plotted on top as black vectors and indicate how each surface marker contributes to the cell variability in a specific direction within the model.
